# Delineation and agreement of FET PET biological volumes in glioblastoma: results of the nuclear medicine credentialing program from the prospective, multi-centre trial evaluating FET PET In Glioblastoma (FIG) study—TROG 18.06

**DOI:** 10.1007/s00259-023-06371-5

**Published:** 2023-08-11

**Authors:** Nathaniel Barry, Roslyn J. Francis, Martin A. Ebert, Eng-Siew Koh, Pejman Rowshanfarzad, Ghulam Mubashar Hassan, Jake Kendrick, Hui K. Gan, Sze T. Lee, Eddie Lau, Bradford A. Moffat, Greg Fitt, Alisha Moore, Paul Thomas, David A. Pattison, Tim Akhurst, Ramin Alipour, Elizabeth L. Thomas, Edward Hsiao, Geoffrey P. Schembri, Peter Lin, Tam Ly, June Yap, Ian Kirkwood, Wilson Vallat, Shahroz Khan, Dayanethee Krishna, Stanley Ngai, Chris Yu, Scott Beuzeville, Tow C. Yeow, Dale Bailey, Olivia Cook, Angela Whitehead, Rachael Dykyj, Alana Rossi, Andrew Grose, Andrew M. Scott

**Affiliations:** 1 School of Physics, Mathematics and Computing, University of Western Australia, WA Crawley, Australia; 2 Centre for Advanced Technologies in Cancer Research (CATCR), WA Perth, Australia; 3 Department of Nuclear Medicine, Sir Charles Gairdner Hospital, Nedlands, WA Australia; 4 Australian Centre for Quantitative Imaging, Medical School, University of Western Australia, Crawley, WA Australia; 5 Department of Radiation Oncology, Sir Charles Gairdner Hospital, Nedlands, WA Australia; 6 Department of Radiation Oncology, Liverpool and Macarthur Cancer Therapy Centres, Liverpool, NSW Australia; 7 South Western Sydney Clinical School, UNSW Medicine, University of New South Wales, Liverpool, NSW Australia; 8 Department of Medical Oncology, Austin Hospital, Melbourne, VIC Australia; 9 Olivia Newton-John Cancer Research Institute, Melbourne, VIC Australia; 10 Department of Medicine, University of Melbourne, Melbourne, VIC Australia; 11 School of Cancer Medicine, La Trobe University, Melbourne, VIC Australia; 12 Department of Molecular Imaging and Therapy, Austin Health, Melbourne, VIC Australia; 13 Department of Radiology, Austin Health, Melbourne, VIC Australia; 14 Department of Radiology, University of Melbourne, Melbourne, VIC Australia; 15 Trans Tasman Radiation Oncology Group (TROG Cancer Research), University of Newcastle, Callaghan, NSW Australia; 16 Department of Nuclear Medicine, Royal Brisbane and Women’s Hospital, Herston, QLD Australia; 17 Faculty of Medicine, University of Queensland, St Lucia, QLD Australia; 18 The Sir Peter MacCallum Department of Oncology, Melbourne, VIC Australia; 19 Department of Nuclear Medicine, Royal North Shore Hospital, St Leonards, NSW Australia; 20 Department of Nuclear Medicine, Liverpool Hospital, Liverpool, NSW Australia; 21 Department of Nuclear Medicine, Royal Adelaide Hospital, Adelaide, SA Australia; 22 Faculty of Health and Medical Sciences, The University of Adelaide, Adelaide, SA Australia; 23 Department of Nuclear Medicine, Canberra Hospital, Woden, ACT Australia; 24 Department of Nuclear Medicine, Princess Alexandra Hospital, Woolloongabba, QLD Australia; 25 Department of Nuclear Medicine, St George Hospital, Kogarah, NSW Australia; 26 Faculty of Medicine 7 Health, University of Sydney, Sydney, NSW Australia

**Keywords:** FET PET, Glioblastoma, Inter-observer, Credentialing, Clinical trials

## Abstract

**Purpose:**

The O-(2-[^18^F]-fluoroethyl)-l-tyrosine (FET) PET in Glioblastoma (FIG) trial is an Australian prospective, multi-centre study evaluating FET PET for glioblastoma patient management. FET PET imaging timepoints are pre-chemoradiotherapy (FET1), 1-month post-chemoradiotherapy (FET2), and at suspected progression (FET3). Before participant recruitment, site nuclear medicine physicians (NMPs) underwent credentialing of FET PET delineation and image interpretation.

**Methods:**

Sites were required to complete contouring and dynamic analysis by ≥ 2 NMPs on benchmarking cases (*n* = 6) assessing biological tumour volume (BTV) delineation (3 × FET1) and image interpretation (3 × FET3). Data was reviewed by experts and violations noted. BTV definition includes tumour-to-background ratio (TBR) threshold of 1.6 with crescent-shaped background contour in the contralateral normal brain. Recurrence/pseudoprogression interpretation (FET3) required assessment of maximum TBR (TBR_max_), dynamic analysis (time activity curve [TAC] type, time to peak), and qualitative assessment. Intraclass correlation coefficient (ICC) assessed volume agreement, coefficient of variation (CoV) compared maximum/mean TBR (TBR_max_/TBR_mean_) across cases, and pairwise analysis assessed spatial (Dice similarity coefficient [DSC]) and boundary agreement (Hausdorff distance [HD], mean absolute surface distance [MASD]).

**Results:**

Data was accrued from 21 NMPs (10 centres, *n* ≥ 2 each) and 20 underwent review. The initial pass rate was 93/119 (78.2%) and 27/30 requested resubmissions were completed. Violations were found in 25/72 (34.7%; 13/12 minor/major) of FET1 and 22/74 (29.7%; 14/8 minor/major) of FET3 reports. The primary reasons for resubmission were as follows: BTV over-contour (15/30, 50.0%), background placement (8/30, 26.7%), TAC classification (9/30, 30.0%), and image interpretation (7/30, 23.3%). CoV median and range for BTV, TBR_max_, and TBR_mean_ were 21.53% (12.00–30.10%), 5.89% (5.01–6.68%), and 5.01% (3.37–6.34%), respectively. BTV agreement was moderate to excellent (ICC = 0.82; 95% CI, 0.63–0.97) with good spatial (DSC = 0.84 ± 0.09) and boundary (HD = 15.78 ± 8.30 mm; MASD = 1.47 ± 1.36 mm) agreement.

**Conclusion:**

The FIG study credentialing program has increased expertise across study sites. TBR_max_ and TBR_mean_ were robust, with considerable variability in BTV delineation and image interpretation observed.

**Supplementary information:**

The online version contains supplementary material available at 10.1007/s00259-023-06371-5.

## Introduction

Glioblastoma (GBM) is the most common primary brain malignancy and has an exceptionally poor prognosis. Those who undergo a maximal tumour resection followed by combined radiochemotherapy, as per standard of care, have a 5-year survival rate of less than 10% [1]. The excellent soft tissue contrast of magnetic resonance imaging (MRI) is routinely used clinically to assist in GBM diagnosis, treatment planning, and response assessment [2, 3]. In the post treatment setting, however, MRI is associated with a lower specificity in identifying neoplastic lesions, limiting its diagnostic power [4]. Advanced MRI sequences such as perfusion and diffusion-weighted imaging may also be used, but the routine clinical availability of these sequences can vary [3, 5, 6].

Positron emission tomography (PET) paired with amino acid tracers has seen increased utilisation for clinical management of GBM. In particular, O-(2-[^18^F]-fluoroethyl)-l-tyrosine (FET) PET imaging has shown promise, due to its ideal half-life (110 min) and imaging characteristics. This includes superior tumour-to-background contrast when compared to the commonly used glucose analogue [^18^F]-2-fluoro-2-deoxy-d-glucose (FDG) PET [7]. Emerging literature has shown that combined FET PET and MRI has increased performance in primary/differential diagnosis compared to MRI alone [8–10]. For treatment planning, volumes derived from FET PET are typically larger and often encapsulate the respective MR-derived gross tumour volume. Although dose escalation based on FET PET has not shown survival benefit, its complimentary use in defining tumour burden has been well investigated [11–15].

Contrast enhancement on MRI depends on disruption of the blood–brain barrier; therefore, assessment of progressive disease is difficult when this same phenomenon can be caused by patient treatment. Since FET uptake within the brain does not require disruption of the blood–brain barrier, it has been shown to supplement the lack of specificity on contrast-enhanced T1-weighted MRI (T1c) when attempting to differentiate tumour recurrence from pseudoprogression or treatment-related change [16–20]. Quantitative FET PET static and dynamic metrics, including relative change in these metrics from baseline to follow-up, have exhibited significant prognostic power [21–25]. Furthermore, the Response Assessment in Neuro-Oncology (RANO) Working Group has advocated for the supplementary clinical use of PET imaging in glioma and brain metastasis [4, 26]. To date, the published literature has been limited to small, single centre retrospective and prospective studies. Since FET PET is likely to play a major role in glioma management in the future, its impact will need to be evaluated across multiple centres.

The O-(2-[^18^F]-fluoroethyl)-l-tyrosine (FET) PET in Glioblastoma (FIG) trial is an Australian, prospective, multi-centre study designed to evaluate the impact of FET PET imaging in GBM management [27]. There are two primary objectives of the FIG study: to investigate how the addition of FET PET imaging to standard MRI imaging affects radiation target volume delineation and treatment planning for GBM, and to determine the accuracy and management impact of FET PET in distinguishing pseudoprogression from true tumour progression and/or tumour recurrence. This study is actively recruiting participants that will undergo FET PET imaging at three timepoints of interest: pre-chemoradiotherapy (FET1), 1-month post-chemoradiotherapy (FET2), and at suspected progression (FET3). Prior to enrolling participants into the prospective phase, each recruiting centre was required to undergo pre-trial quality assurance consisting of an initial credentialing phase. The nuclear medicine physician (NMP) contour data resulting from this credentialing phase have been incorporated into a study on inter-observer variability. Here, we summarise and assess NMP performance on image analysis at FET1 and FET3 as part of credentialing, with further quantitative investigation of variability in the following: volumetric assessment with FET PET, conventional quantitative metrics extracted from these volumes, background assessment, spatial overlap, and boundary agreement.

## Methods and materials

### Credentialing dataset

Six anonymised, representative FET PET scans, acquired with standardised protocols, were used for the credentialing study. Three FET scans were post-surgical delineation cases (FET1CASE1, FET1CASE2, and FET1CASE3, respectively) and three FET scans were obtained at suspected progression (FET3CASE1, FET3CASE2, and FET3CASE3, respectively). The six cases were representative of typical clinical cases for each scenario and were distinct in terms of intensity of uptake, location, biological tumour volume (BTV), and diagnosis. Relevant clinical information was available for each case (Table 1) and an instruction manual was provided to all NMPs who were performing the analysis (Supplementary Material 1). In accordance with the FIG study, imaging at FET2 is not directly assessed. It is, however, made available as previous imaging, when assessing treatment response at FET3. As such, this timepoint was not included for credentialing.

**Table 1:** Clinical characteristics of the six nuclear medicine credentialing cases

Case	Sex/age	Localisation	Treatment^a^
FET1CASE1	F, 58	Right frontoparietal	Partial resection
FET1CASE2	M, 67	Right temporal	Gross total resection
FET1CASE3	F, 60	Left frontoparietal	Resection^b^
FET3CASE1	F, 55	Left parietal	Subtotal resection + 60 Gy/30 fractions + veliparib^c^/temozolomide
FET3CASE2	M, 37	Left parietal	Near gross total resection + 60 Gy/30 fractions + temozolomide
FET3CASE3	M, 59	Right temporoparietal	Near gross total resection + 60 Gy/30 fractions + temozolomide

*N/A* not available, *M* male, *F* female

^a^Completed at time of imaging

^b^Extent not specified

^c^Patient from the VERTU trial (ACTRN12615000407594)

### Image acquisition/details

The credentialing scans were obtained from two sites, using a standardised imaging protocol. Images from the FET1 cases were taken from Sir Charles Gairdner Hospital, Western Australia (Human Research Ethics Committee approved study 2014–004). Images from the FET3 cases were taken from Royal North Shore Hospital, New South Wales (all subjects gave written informed consent for their data to be used). Patients were required to fast for a minimum of 4 h prior to imaging. Scans were taken, following intravenous administration of 200 MBq of FET, on a Biograph 16 PET/CT, Siemens (CTI Inc., Knoxville, TN). A low-dose CT was performed, immediately after administration, for attenuation correction. A dynamic acquisition followed (FET1 30-min acquisition, FET3 40-min acquisition) with the final static image consisting of the summed PET data (FET1 20–30 min, FET3 20–40 min) post-injection of tracer. Dead time, attenuation, scatter, decay, and random corrections were applied, along with detector normalisation. Iterative reconstruction for the FET1 cases was performed with point-spread function (PSF) applied (TrueX): 3 iterations, 24 subsets, matrix size = 168 × 168, zoom factor = 2, post-reconstruction filter = 4 mm full width at half maximum (FWHM) Gaussian kernel. Images were reconstructed to a voxel spacing of 2.03135 × 2.03135 × 3 mm^3^. Iterative reconstruction for the FET3 cases was performed with PSF (TrueX), and time-of-flight (TOF) applied: 2 iterations, 21 subsets, matrix size = 400 × 400, zoom factor = 2, post-reconstruction filter = 2 mm FWHM Gaussian kernel. Images were reconstructed to a voxel spacing of 2.03642 × 2.03642 × 3 mm^3^.

### Contouring protocol and dynamic analysis

At least two NMPs from each of the 10 selected study sites completed BTV delineation on all six cases. NMPs were required to delineate each case in accordance with the FIG study imaging and radiotherapy quality assurance manual as per the study protocol and using a MiM workflow developed for the FIG study (MiM Encore version 7.0, MiM Software Inc., Cleveland, OH). Briefly, delineation of the BTV followed a semi-automatic procedure. Background assessment was achieved by placing a crescent-shaped volume of interest (VOI), including grey and white matter, in the hemisphere contralateral to the suspected lesion [28]. Background is defined as the mean standardised uptake value (SUV), SUV_mean_, for that VOI. Amino acid uptake in the BTV was defined using a 1.6 tumour-to-background ratio (TBR) threshold on a spherical VOI (Static VOI) placed around the suspected tumour [29]. The segmented volume was then manually adjusted, if required, to remove any obvious non-tumour structures (e.g. scalp, sinus uptake). For the purposes of this study, the volume obtained after thresholding, but prior to manual adjustment, was referred to as GTV0. The final volume is referred to as the BTV. TBR_max_ was calculated by dividing the maximum SUV (SUV_max_) of the BTV by SUV_mean_ of the background and TBR_mean_ was calculated by dividing SUV_mean_ of the BTV by SUV_mean_ of the background. Lastly, as part of the dynamic analysis, a 1-mL spherical VOI was automatically generated and centred on SUV_max_ in the BTV to create a time activity curve (TAC) from the dynamic data. Time to peak (TTP) was calculated and NMPs were instructed to classify TACs into three types for each case, as described previously [30].

### Credentialing and analysis of violations

Each set of structures and dynamic analysis was submitted to the Trans-Tasman Radiation Oncology Group (TROG) for expert NMP review. The NMPs from each site had a range of experience in FET PET review and analysis. The 21 NMPs involved in the credentialing program were all experienced, with four having 5–9 years of clinical experience and 17 having over 10 years of clinical experience. In contrast, there was a wide range of NMP prior familiarity with FET PET analysis and interpretation, with seven NMPs having zero experience, eight having minimal experience, two having moderate experience, and four having significant experience. Expert review of each report was performed by one of three NM specialists (STL, RJF, EL) with clinical experience in FET PET contouring, interpretation, and analysis. As part of credentialing, protocol compliance was assessed as acceptable, minor, or major based on BTV delineation (FET1 cases) and response interpretation (FET3 cases). Interpretation was informed by an initial qualitative assessment, which was reported descriptively as either suggestive of tumour progression or favouring treatment predominant changes. In conjunction, quantitative parameters were calculated from the FET3 cases, including TBR_max_, TTP, and TAC type [17]. Both were combined to inform clinical response interpretation as either treatment predominant changes, equivocal, or consistent with tumour progression. In clinical practice, comparison with previous findings is crucial to interpretation, which will be conducted in the FIG study. However, no available study matched the design of the FIG study when developing the credentialing dataset; hence, previous imaging (i.e. FET1 and FET2) was not available to NMPs when evaluating the FET3 cases. NMPs were asked to resubmit to address major violations in protocol compliance. The rate and reason for minor/major violations were documented and resubmission rate was recorded. The expert comments provided to each NMP were then processed and analysed to assess the frequency of violation reasons. As part of this analysis, violation reasons were categorised and defined as follows: (1) BTV under-contouring where the NMP has failed to include important areas of uptake, (2) BTV over-contouring where the NMP has applied an unnecessary manual increase of the BTV and/or failed to remove non-tumour structures after thresholding, (3) incorrect background placement on the white–grey matter junction, (4) incorrect classification of TACs into type I, II, or III, and (5) image interpretation not concordant with reviewer.

### Statistical analysis

Descriptive statistics are reported as the mean/standard deviation, median/range, and coefficient of variation (CoV). The CoV is a normalised measure of variability to compare metrics between cases with differing means (CoV = standard deviation/mean). Delineated structures were exported from MiM in DICOM RTStruct format and converted to NIfTI binary masks using Plastimatch, an open-source software for image computation.^1^ To assess volume overlap, pairwise analysis was conducted between each NMP per case, measured using the Dice similarity coefficient (DSC). Furthermore, since DSC does not assess boundary differences, pairwise Hausdorff distance (HD) and mean absolute surface distance (MASD) were calculated. This combination of metrics adequately assesses contouring variations required for this analysis [31]. The pairwise analysis was computed using PlatiPy.^2^ Lastly, the intraclass correlation coefficient (ICC) is calculated to assess inter-observer reliability of contours using a two-way mixed model (absolute agreement, single rater/measurement) [32, 33]:

$$\mathrm{ICC}\left(\mathrm{2,1}\right)=\frac{M{S}_{R}-M{S}_{E}}{M{S}_{R}+\left(k-1\right)M{S}_{E}+\frac{k}{n}(M{S}_{C}-M{S}_{E})}$$

where *MS*_*R*_ is the mean square for rows (between subjects), *MS*_*E*_ is the mean square for error, *MS*_*C*_ is the mean square for columns (between raters/measurements), *n* is the number of subjects, and *k* is the number of raters/measurements. The ICC ranges from 0 to 1, with 1 being perfect reliability. Here, the ICC is interpreted based on the 95% confidence intervals (CI below 0.5 is poor; 0.5 to 0.75 is moderate; 0.75 to 0.9 is good; above 0.9 is excellent), as per recommendations of Koo and Li [34]. The ICC was calculated in Python using the psych package (version 2.2.5) from R. The rpy2^3^ interface was implemented to run R within a Python process.

## Results

**Fig. 1:**
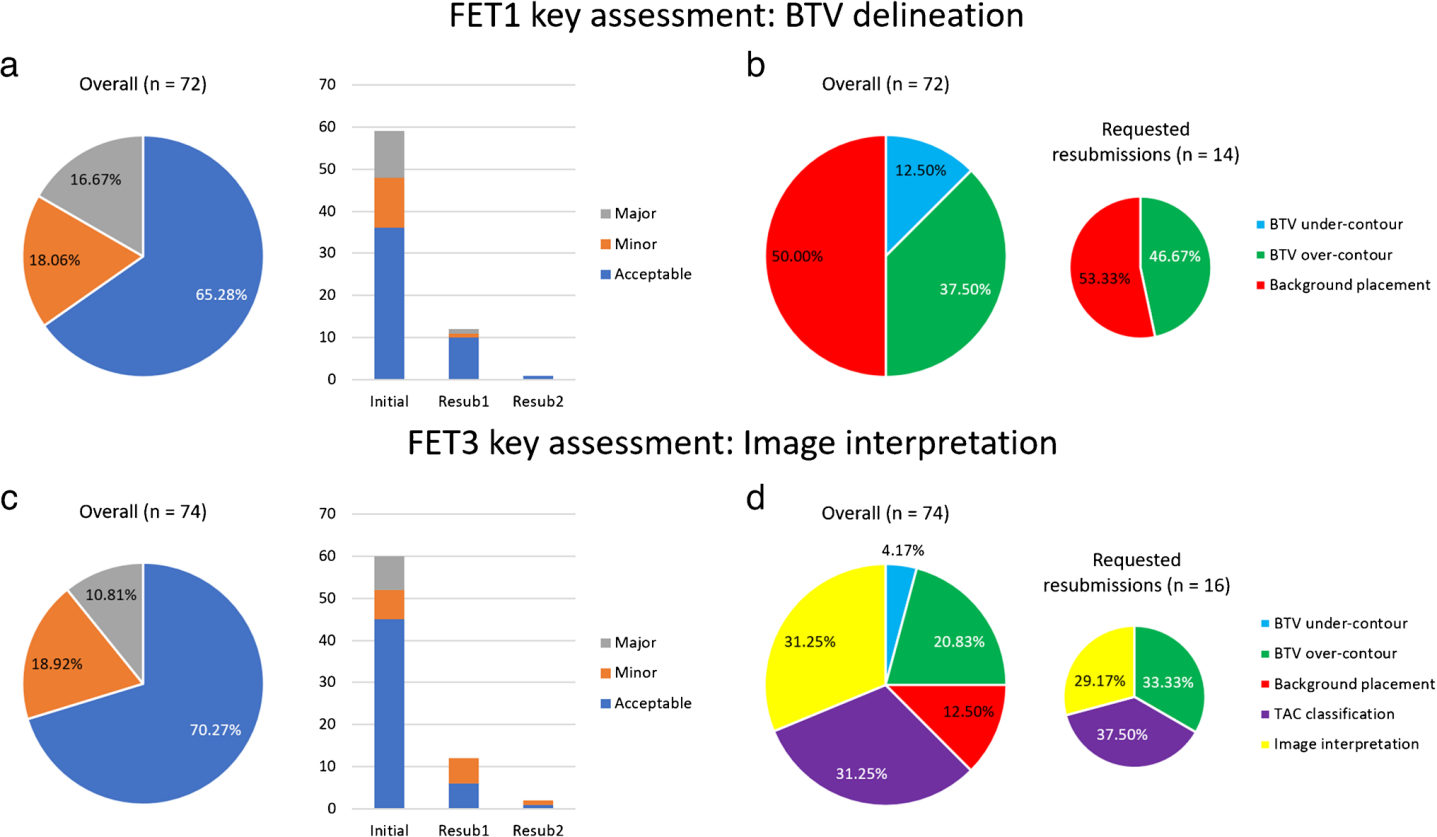
An overview of NMP credentialing by expert reviewers and their sources of violations of the two key assessments during credentialing: FET1 BTV delineation and FET3 image interpretation. The frequency of acceptable, minor, and major violations is shown for FET1 (**a**) and FET3 (**c**), with violations split into initial, first resubmission, and second resubmission. Frequency of FET1 (**b**) and FET3 (**d**) violation reasons, accrued from reviewer comments, is shown. Violation reasons are also broken down into reports that requested resubmissions to highlight NMP critical errors

Twenty-one NMPs from 10 centres submitted data for credentialing. One expert did not undergo review and a reporting physician contoured FET1CASE1 instead of FET1CASE3 in one instance which was not discovered during review and was subsequently passed. This incorrect submission was not included in further analysis. In total, 146 case reviews were conducted which included 119 initial submissions and an additional 27/30 resubmissions. The reports of three resubmissions were incomplete at the time of analysis. In three instances, two resubmissions were required. Reporting NMPs with zero or minimal prior experience with FET PET accounted for 26/30 (86.7%) requested resubmissions. The resulting performance of NMPs and recorded violations are shown in Fig. 1. Overall, a pass rate of 93/119 (78.2%) on the initial submissions was observed, with some form of violation in 25/72 (34.7%; 13 minor/12 major) of the FET1 submissions and 22/74 (29.7%; 14 minor/8 major) of the FET3 submissions. Overall, the 30 resubmissions that were requested by the expert reviewers were often triggered by either BTV over-contouring (15/30, 50.0%), an error in background placement (8/30, 26.7%), an error in TAC classification (9/30, 30.0%), or image interpretation not concordant with expert (7/30, 23.3%). It should be noted that these sources of violation are not mutually exclusive and there are some instances where reviewers provided multiple reasons when giving feedback to reporting NMPs. A summary of the case reviews can be found in Supplementary Material 2 Table S1.

**Fig. 2:**
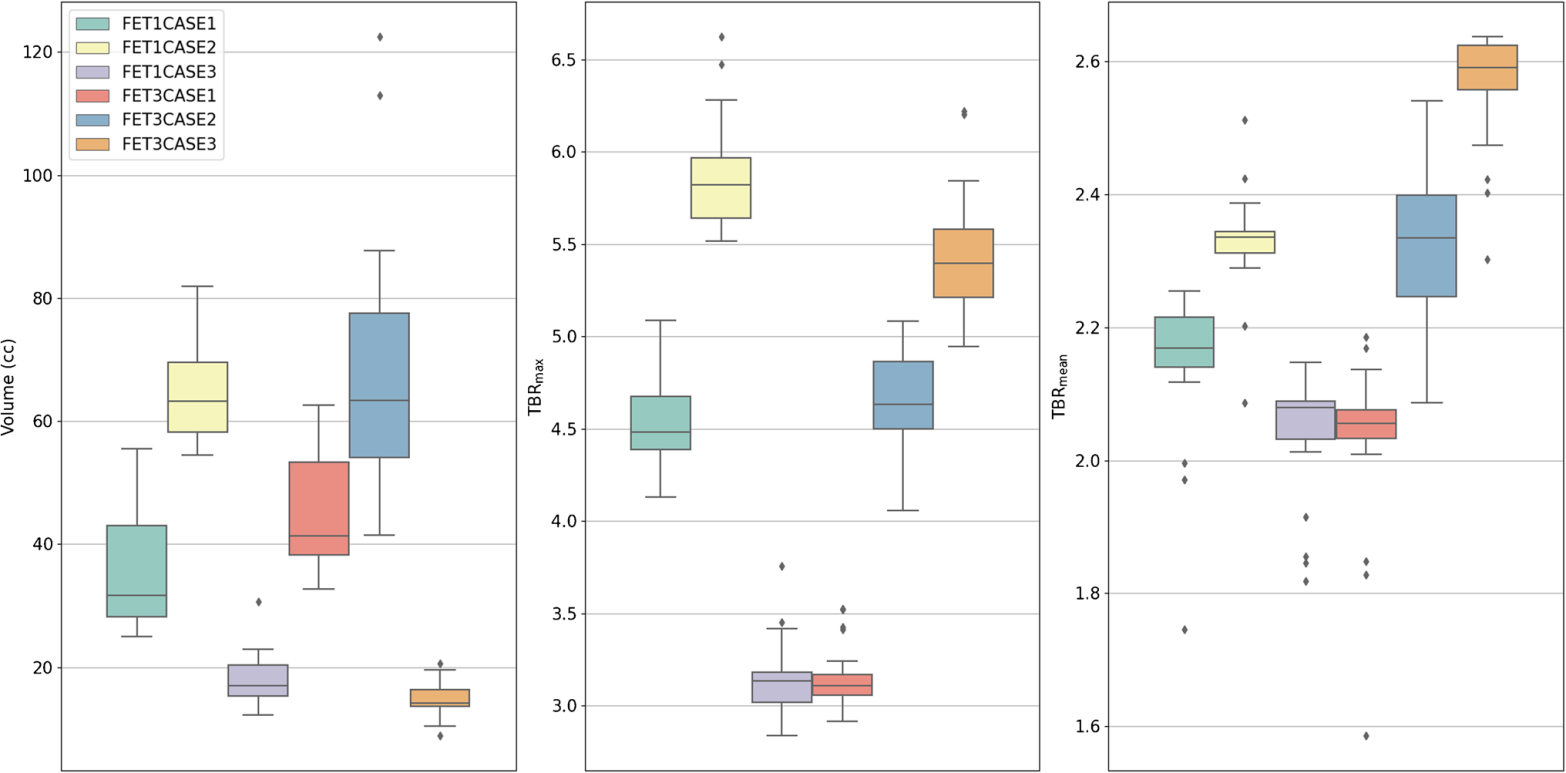
Boxplots showing the distribution of NMP BTVs (left), TBR_max_ (middle), and TBR_mean_ (right) grouped by each nuclear medicine credentialing case

For the quantitative assessment of NMP agreement, 125/126 structure sets (BTV, GTV0, Background, Static VOI) were available for analysis. If an NMP resubmitted contours, their final submission was used. All calculated metrics for the BTV are shown in Table 2. Variability of the BTV gave a median CoV of 21.53% (range, 12.00–30.10%). Variation in BTV delineation was lowest for FET1CASE2 (64.69 ± 7.76 cm^3^) and highest for FET3CASE2 (68.32 ± 20.56 cm^3^). Boxplots visualising the distribution of BTV, TBR_max_, and TBR_mean_ by each case are shown in Fig. 2. Both TBR_max_ (CoV median, 5.89%; range, 5.01–6.68%) and TBR_mean_ (CoV median 5.01%; range, 3.37–6.34%) had similar variability across cases. The ICC was calculated from 20/21 NMPs and inter-observer agreement was found to be moderate to excellent (ICC = 0.82; 95% CI, 0.63–0.97) for contoured BTVs. Furthermore, inter-observer agreement of GTV0 was similar prior to manual adjustment (ICC = 0.82; 95% CI, 0.62–0.96). It should be noted that the CoV of GTV0 compared to the BTV increased in 3/6 of the benchmarking cases, although this increase was not greater than 4% (Supplementary Material 2 Table S2). To assess the impact of the resubmission process, the ICC was calculated on initial contours only, which was found to be poor to excellent (ICC = 0.69; 95% CI, 0.44–0.93). The resubmission process caused a marked increase in the ICC of 0.13 (0.69 to 0.82), with the lower bound of the 95% CI increasing from 0.44 to 0.63 which displays a quantitative improvement in NMP agreement. Contouring of the background resulted in reasonable variability in uptake with SUV_mean_ median CoV = 5.81% (range, 4.78–6.32%).

**Table 2:** Mean, standard deviation (SD), and coefficient of variation (CoV) of BTV, TBR_max_, TBR_mean_, and pairwise DSC, HD, and MASD for the six nuclear medicine credentialing cases of the FIG study

		FET1CASE1	FET1CASE2	FET1CASE3	FET3CASE1	FET3CASE2	FET3CASE3
Volume	Mean (cm^3^)	34.13	64.69	17.99	44.17	68.32	14.78
	SD (cm^3^)	8.13	7.76	4.29	8.40	20.56	2.84
	CoV (%)	23.83	12.00	23.87	19.01	30.10	19.23
TBR_max_	Mean	4.54	5.86	3.15	3.15	4.67	5.43
	SD	0.29	0.29	0.21	0.17	0.25	0.35
	CoV (%)	6.30	5.01	6.68	5.47	5.40	6.36
TBR_mean_	Mean	2.15	2.33	2.04	2.03	2.32	2.56
	SD	0.11	0.08	0.10	0.13	0.12	0.09
	CoV (%)	5.29	3.38	4.73	6.34	5.33	3.37
DSC	Mean	0.85	0.88	0.85	0.85	0.77	0.84
	SD	0.08	0.05	0.09	0.08	0.09	0.08
	CoV (%)	9.72	5.36	10.29	9.03	11.64	9.70
HD	Mean (mm)	15.14	12.78	10.15	14.59	23.37	18.09
	SD (mm)	7.84	2.74	5.65	6.25	9.40	8.89
	CoV (%)	51.76	21.45	55.69	42.81	40.22	49.14
MASD	Mean (mm)	1.24	0.77	1.09	1.10	3.41	1.20
	SD (mm)	0.82	0.34	0.78	0.68	1.95	0.77
	CoV (%)	65.62	44.55	71.21	62.01	57.32	63.89

**Fig. 3:**
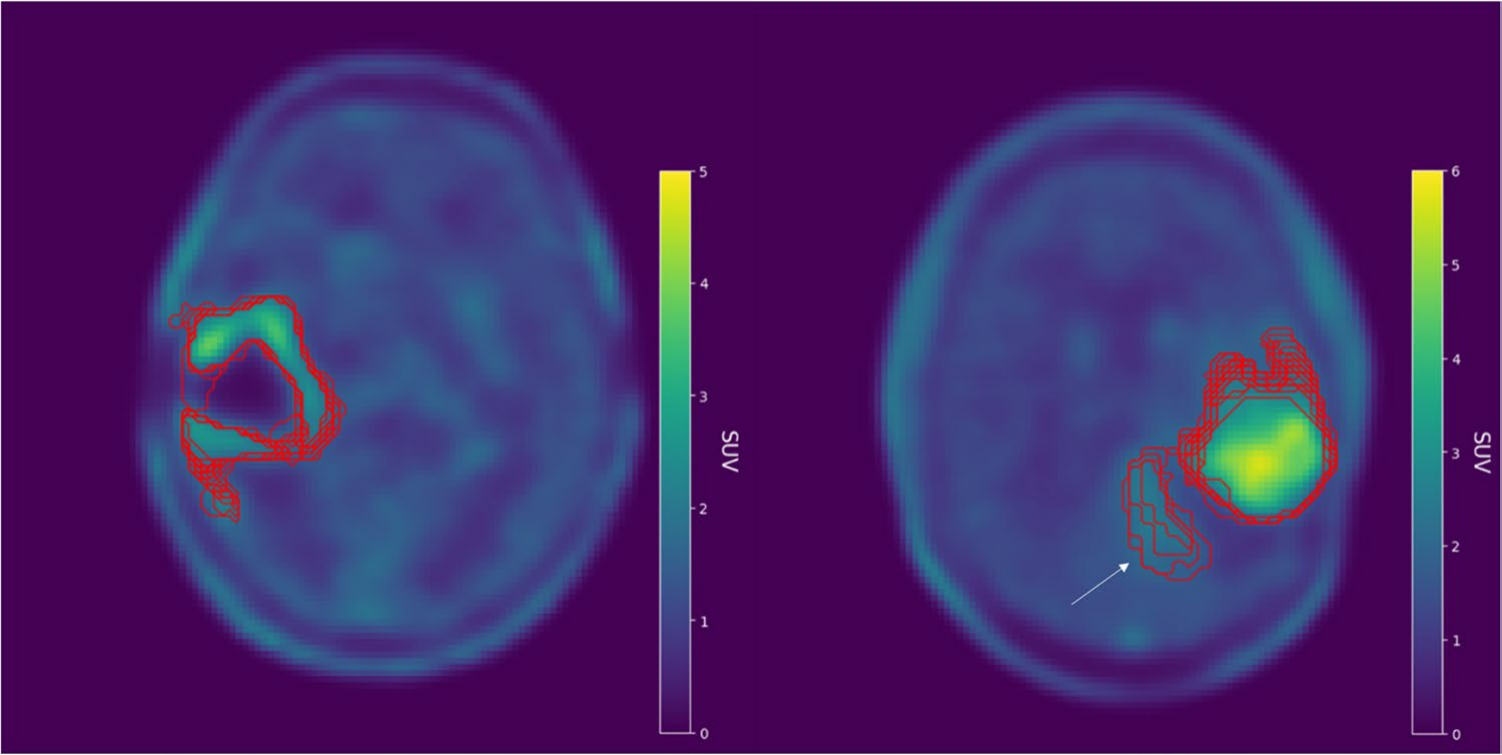
Illustration of credentialing image FET1CASE2 that exhibited the best (DSC = 0.88 ± 0.05) agreement amongst NMPs (left) and credentialing image FET3CASE2 that showed the poorest (DSC = 0.77 ± 0.09) agreement (right). Superimposed NMP contours are shown in red. Discrepancy between NMPs when including the additional area of uptake (white arrow) in FET3CASE2 is likely the main source of variability in the pairwise analysis

**Fig. 4:**
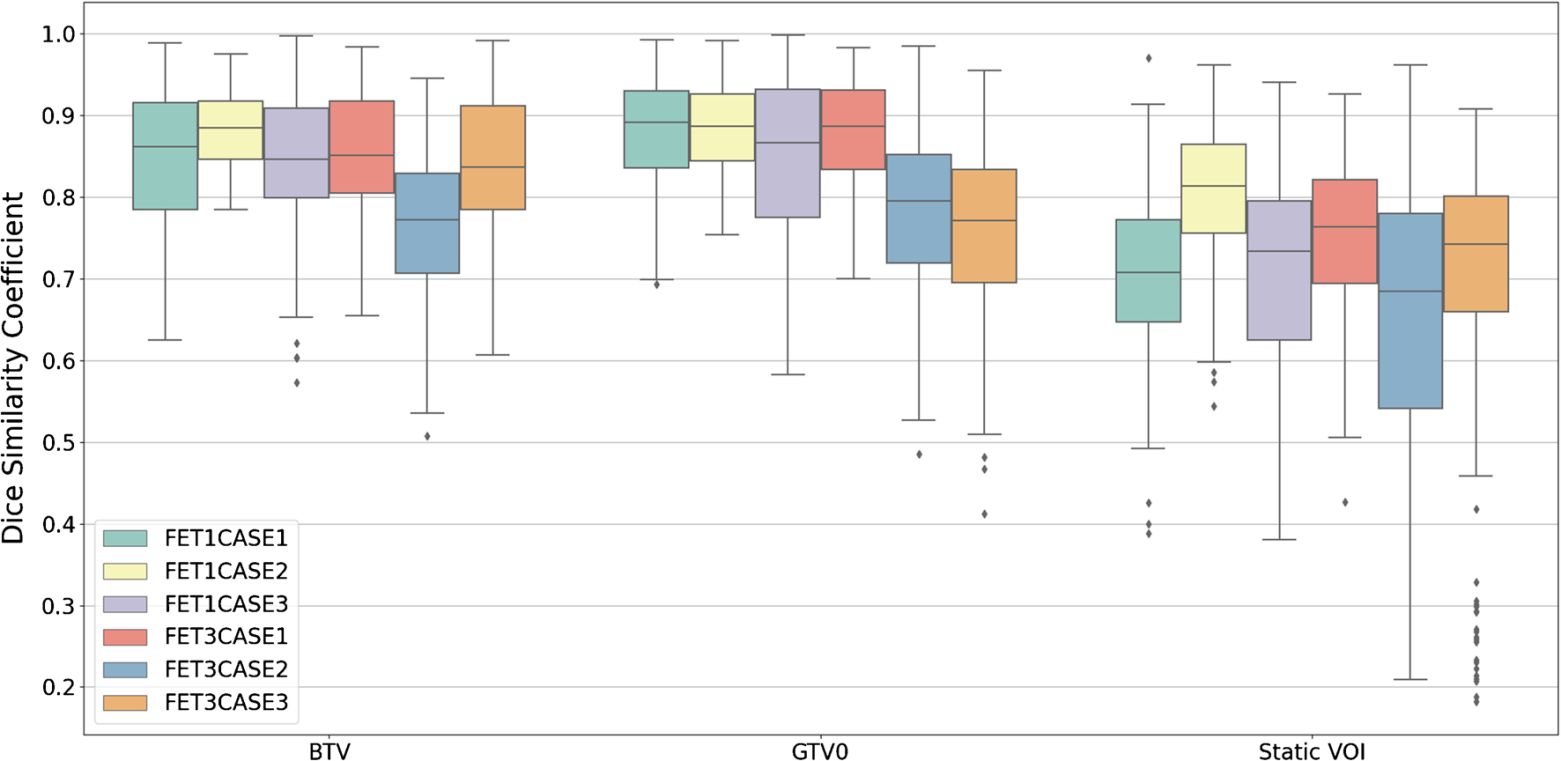
Boxplots visualising the distribution of pairwise Dice similarity coefficient (DSC) of each delineated structure, grouped by case. The biological tumour volume (BTV), GTV0 (volume obtained after threshold is applied), and Static VOI are all generated as part of the delineation process. For each set of credentialing cases, spatial overlap is assessed by calculating the DSC for every pairwise combination of NMP contours

Pairwise analysis for the BTV revealed good overlap agreement across all cases (DSC = 0.84 ± 0.09), with every case reporting a mean DSC > 0.8, except for FET3CASE2 (DSC = 0.77 ± 0.09). HD was on average > 10 mm for each case, with FET3CASE2 exceeding 20 mm (HD = 15.78 ± 8.30 mm across all cases), reaching over 40 mm in some pairwise comparisons. Although, HD is sensitive to outliers and the presence of small discrepant islands of uptake in the delineated BTV can increase pairwise distance drastically. However, average boundary differences revealed that they rarely exceeded 3 mm, with MASD = 1.47 ± 1.36 mm exhibited across all cases. FET3CASE2 showed the greatest separation of boundaries with MASD = 3.41 ± 1.95 mm. Overall, pairwise comparison shows that BTV analysis of FET1CASE2 demonstrated the best agreement, whereas FET3CASE2 had the worst (Fig. 3). Data relating to DSC, HD, MASD, and background SUV_mean_ of all NMP contours can be found in Fig. 4 and in the supplementary material (Supplementary Material 2, Tables S3–S6, Figs. S1–S3).

## Discussion

The increased utilisation of FET PET and the incorporation of semi-quantitative parameters for clinical evaluation have highlighted the need for a greater understanding of how NMPs interpret FET PET studies. Accurate tumour delineation and interpretation of FET PET are crucial for glioblastoma patient management, which includes assessment of newly diagnosed tumour for biopsy planning and surgical intervention, differential diagnosis at suspected progression, and treatment monitoring. The most frequent indication for FET PET scans being performed is at suspected tumour recurrence [35]. Although clinical evaluation primarily evaluates qualitative parameters and TBRs, the BTV also plays a role as a prognostic biomarker and for treatment response when comparing previous imaging timepoints [36, 37]. It is further useful to assess the clinical role of the BTV in radiotherapy planning and monitoring of glioma patients, as recently reviewed by the PET/RANO group [38].

We have performed the largest reported FET PET credentialing study, incorporating 21 NM specialists, across 10 different centres each undertaking analysis of 6 scans (126 total) to assess reporting performance and contour variability. The expert review and resubmission process demonstrated that the majority (86.7%) of significant errors were made by NMPs with no or minimal experience in FET PET studies and highlighted several key areas that required education and training, including manual adjustment of the final BTV, the adequate encapsulation of the white–grey matter junction in the background region, TAC type classification, and FET3 image interpretation. The BTV variability exhibited in this credentialing study was moderate to excellent as assessed by the ICC, with good pairwise comparison between NMPs when assessing spatial overlap and boundary differences.

Both BTV over-contouring and background placement were primary reasons for FET1 violations and resubmissions. BTV under-contouring was mentioned in 12.5% of violations but was not present in any report where a resubmission was requested. Given the contouring protocol, under-contouring will most often occur when placing the initial sphere, which never resulted in a major violation. However, BTV over-contouring was far more common and was mentioned a total of 20 times across both FET1 and FET3 cases, usually resulting from unnecessary manual adjustments, nine of which mentioned NMPs failing to remove scalp uptake. Key assessment for the FET3 cases was interpretation, with error in TAC classification and image interpretation not concordant with expert being the primary sources of violations. Background violations were not mentioned in any FET3 reports where a resubmission was requested. Perhaps, since an error in background would directly impact TBR_max_, it was not to a degree that interpretation would be significantly impacted. To further this point, an important threshold for interpretation is TBR_max_ > 2.3 which no NMP fell below, as shown in Fig. 2 [17]. It should be noted that correct BTV delineation was not a strictly assessed criterion in the FET3 cases as it was in the FET1 cases, although correct delineation is important for the FIG study as NMPs will compare with previous imaging for response assessment and prognostic analysis. As such, there were four instances where an NMP’s interpretation was considered acceptable, but their contoured regions were failed by the expert reviewer. Furthermore, although TAC classification and image interpretation were commented on equally when analysing FET3 violations, they occurred together in only six case reports. In many instances, an error when assigning TAC types did not coincide with an error in interpretation and vice versa. Regardless, TAC type misclassifications were a more frequent source of error in FET3 analysis, rather than abnormal SUV_max_/TBR_max_ which was only mentioned by a reviewer once, with another resulting from a technical error related to the dataset rather than a user error.

In an inter-observer study with 30 newly diagnosed high-grade glioma (HGG) cases, DSC overlap of FET PET was found to be 0.922 (95% CI, 0.910–0.934), with excellent volume agreement (ICC = 0.986; 95% CI, 0.975–0.993) [39]. Excellent agreement was also found segmenting on T1c (ICC = 0.969; 95%CI, 0.944–0.984) and good to excellent on T2-FLAIR (ICC = 0.929; 95% CI, 0.871–0.964). A 1.6 TBR threshold was applied to a 30-mm margin around the MRI-based GTV, whereas, in this study, a spherical VOI was placed around the suspected tumour volume. The placement of the initial spherical VOI was an overt source of variability found in the contours of FET3CASE2, as the initial sphere placed by NMPs covered the additional area of uptake to varying degrees (Supplementary Material 2 Fig. S4). Furthermore, a study using 40% SUV_max_ to delineate the BTV reported excellent inter-observer agreement (ICC = 0.9; no CIs given) across 19 post-operative HGG cases [40]. Lastly, in a study of 3,4-dihydroxy-6-[^18^F]-fluoro-l-phenylalanine (FDOPA) PET of 19 HGG cases, the mean Jaccard index of PET inter-observer volume overlap was 0.42 ± 0.22 [41]. As this is equivalent to a mean DSC of 0.59, the lower overlap agreement when compared to this study is likely due to the manual FDOPA PET delineation methodology.

Variability of TBR_max_ and TBR_mean_ was similar as measured by the CoV, although TBR_mean_ exhibited a noticeable number of outlier values which warranted further investigation. Outliers far below the median in each case were caused by a manual addition of volume to GTV0 after thresholding to give the final BTV. In a particular instance, an NMP returned a TBR_mean_ < 1.6 when assessing FET3CASE1, which was due to the manual inclusion of a central area of low activity/necrosis. It is expected that several outliers would be avoided if NMPs were restricted from adding more volume to GTV0 when finalising the BTV, as this approach is likely to include voxels with a TBR < 1.6. Finally, outliers far above the median in each case tended to coincide with background assessments that had relatively low SUV_mean_, although this occurred less frequently (Supplementary Material 2 Fig. S5).

A background measurement that is robust to intra- and inter-observer variability is key to adequately delineate a volume that separates malignant uptake from normal uptake within the brain. Furthermore, variability in TBR_max_ typically relies on the mean background activity acquired as we found that all NMPs obtained the same SUV_max_ within their delineated BTVs. Although, there may be a small possibility, in certain cases, where SUV_max_ is peripheral to the central tumour area, which could be missed when applying the initial sphere. The crescent-shaped volume used in this study for background assessment is based on the work by Unterrainer et al. (2017) [28] which reported SUV_mean_ median CoV of 2.14% (range, 1.05–7.23%) evaluated by six readers across 20 scans. In this study, the SUV_mean_ median CoV was higher at 5.81%, although the range of values reported here (4.78–6.32%) is within said range. It should be noted that the methodology in drawing the crescent-shaped volume reported by Unterrainer et al. differed when compared to the FIG workflow. In the FIG workflow, NMPs are only required to draw the background region of interest on one slice, which then generates a 3D cylindrical “tube” as the crescent-shape is drawn (Supplementary Material 2 Fig. S6), whereas the methodology by Unterrainer et al. involved the drawing of a crescent-shaped ROI on six consecutive axial slices which were fused to form the VOI. This is perhaps a source of difference in the inter-observer variability reported here. Another study has also reported a methodology for background assessment using a semi-automatic generation of mirror-image reference regions, assessed on recurrent GBM patients, to remove arbitrary volume definitions that are part of the delineation process [42]. Reported inter-observer variability in background SUV_mean_ when using a guided crescent-shape approach was also lower (median CoV 2.80%; range, 1.00–4.35%), but variability for the BTV (median CoV 14.37%; range, 5.03–36.30%) was not far from that reported in this study. Furthermore, automatic lesion detection of gliomas has been explored using a 3D U-Net [43], with reported DSC up to 0.8231 on the validation set, similar to the level of mean inter-observer overlap found in this study. As a potential approach, the automatic placement of the initial spherical VOI around the tumour volume and background contour could be investigated, with an NMP making manual adjustment where needed, to streamline the clinical workflow.

In comparison to PET, studies have also assessed inter-observer agreement in the delineation of gliomas on MRI. In a longitudinal study of inter-observer agreement, expert segmentation of GBM enhancing tumour elements on T1c was excellent for pre-operative MRI, good to excellent for post-operative MRI, although with low spatial overlap, and good to excellent agreement on MRI at progression [44]. A definitive decrease in volume agreement was observed for non-enhancing GBM tumour elements segmented on T2/FLAIR images in the post-operative setting, reporting poor to moderate agreement. Visser et al. (2019) [44] noted that agreement on absence of residual enhancing tumour on post-operative MRI in several patients may have contributed to a high ICC. Additionally, a study of 8 GBM cases on delineating enhancing tumour on T1c exhibited excellent agreement on pre-operative MRI and poor to good agreement on post-operative MRI [45]. Lastly, mean DSC was found to be ≥ 0.93 when assessing intra-observer, inter-observer, and between software semi-automatic segmentation agreements, in a study of 18 pre-operative GBM cases [46]. Structural MRI combined with the physiological information provided by FET PET may help to improve spatial overlap of physician contours, particularly in the post-operative setting.

One potential limitation of the credentialing process is the relatively small number of patient cases that were evaluated. The large number of reporting NMPs and participating centres would have made the inclusion of more cases for qualitative and semi-quantitative analysis impractical. However, from the credentialing program, a workflow has been developed that can be used in a potential audit on a larger number of live cases following completion of the FIG study. Although the cohort was small and included only GBM cases, the choice of FET scans used for credentialing ranged in terms of lesion location, extent of uptake, tumour size, and diagnosis. There is a possibility that the imaging manual provided for NMPs who performed the analysis was not sufficiently detailed, leading to the errors in interpretation, although feedback from the central expert reviewers was able to correct these errors in subsequent analysis. The development of more detailed instruction manuals, or training videos, may address this important point in larger studies or for routine clinical implementation of FET PET studies. There were slight differences in time of acquisition and image reconstruction between the FET1 and FET3 sets of cases; however, we do not believe this substantially impacted on the interpretation of the cases used. Furthermore, axial T2 or FLAIR alongside axial T1c was available in the FET1 cases, whereas only axial T1c was available in the FET3 cases. Sources of discrepancy between this study and others may include that, upon expert review, some NMP contours may have had errors that were noted but were not extensive enough to cause a resubmission, which would have reduced agreement. Our study also used a 1.6 TBR threshold which was established in untreated gliomas, and whilst it is commonly used in FET PET interpretation, an alternative threshold of 1.7–1.8 has been proposed for the assessment of relapsed GBM [47]. Although no other study reported a review and resubmission process, it was a necessary part of credentialing, as some NMPs from participating centres were unfamiliar with FET PET contouring (majority with zero/minimal prior experience). This process identified sources of NMP deviation from protocol when contouring on FET PET, across all participating centres, which could be corrected prior to patient recruitment as part of the prospective phase.

## Conclusion

The FIG credentialing program was undertaken to evaluate NMP variability in FET PET lesion delineation, and to increase expertise and standardisation of semi-quantitative analysis, enabling sites participating in the FIG study across multiple centres to commence recruitment. BTV volume agreement was found to be moderate to excellent as assessed by the ICC. Pairwise analysis revealed good spatial overlap and boundary agreement across all cases, and it was found that placement of the initial spherical VOI can be an unexpected source of NMP disagreement in certain cases. Submissions primarily found sources of violations to result from manual BTV adjustment, placement of the background contour on the white–grey matter junction, TAC type classification, and image interpretation. The resubmission process addressed these sources of major violation which were passed accordingly. These will be important areas of focus in the analysis performed for the recruitment phase of the study.

### Supplementary Information

Below is the link to the electronic supplementary material.

Supplementary file1 (PDF 4430 KB)

Supplementary file2 (DOCX 1226 KB)

## Footnotes

1 http://plastimatch.org/ (version 1.9.0).

2 https://github.com/pyplati/platipy (version 0.4.1).

3 https://rpy2.github.io/ (version 3.5.4).
