# Supplementary material for: Delineation and agreement of FET PET biological volumes in glioblastoma: results of the nuclear medicine credentialing program from the prospective, multi-centre trial evaluating FET PET In Glioblastoma (FIG) study—TROG 18.06
Source: Eur J Nucl Med Mol Imaging. 2023 Aug 11;50(13):3970–81. doi: 10.1007/s00259-023-06371-5 (PMC10611835; doi:10.1007/s00259-023-06371-5)
Supplement: Supplementary file 1 — Supplementary file1 (PDF 4430 KB) [file 259_2023_6371_MOESM1_ESM.pdf]

FIG v1.1 Guide

Prerequisites

- MIM Version: 7.0+ ●
  Settings:
  - Settings->General Preferences->Viewing: Always prompt to confirm workflow series assignments, unchecked
  - Settings->General Preferences->Viewing->SUV: SUV Calculation Type: Body Weight
  - Settings->General Preferences->Viewing->SUV: Minutes from FDG Dose to Scan(Min): 0
  - Settings->General Preferences->Imaging->Contouring->Advanced: Allow SUV Peak to include data outside the contour for small structures
  - Settings->General Preferences->DICOM->Loading: Reorient Non-HFS Axial MR scans to Standard Orientation: Never Reorient
  - Settings->Patient List Options: Use Classic Patient List, checked

Workflow Import

1. Download the “FIG v1.1.zip” workflow file to your computer. No need to unzip it
2. Launch MIM and toggle on the Workflow Panel where you can find the ‘Organize..’ button in the down below,
3. Click the ‘Organize..’ button to bring up the ‘Import Manager’ window,
4. Click the ‘Open file’, the middle button in the upper right corner, to bring up the ‘Open file’ window,
5. Navigate to the folder where you put the workflow file, select the file and open it,
6. Select(Ctrl+A) all the files in the Source listing, or Left, column and hit the ‘Import Selected’ button to import all the files,
7. Hit the ‘Apply’ and ‘Ok’ button to finish the importing process.
8. When the imported workflows in the right column are in yellow conflicted color, you need to overwrite the old ones,

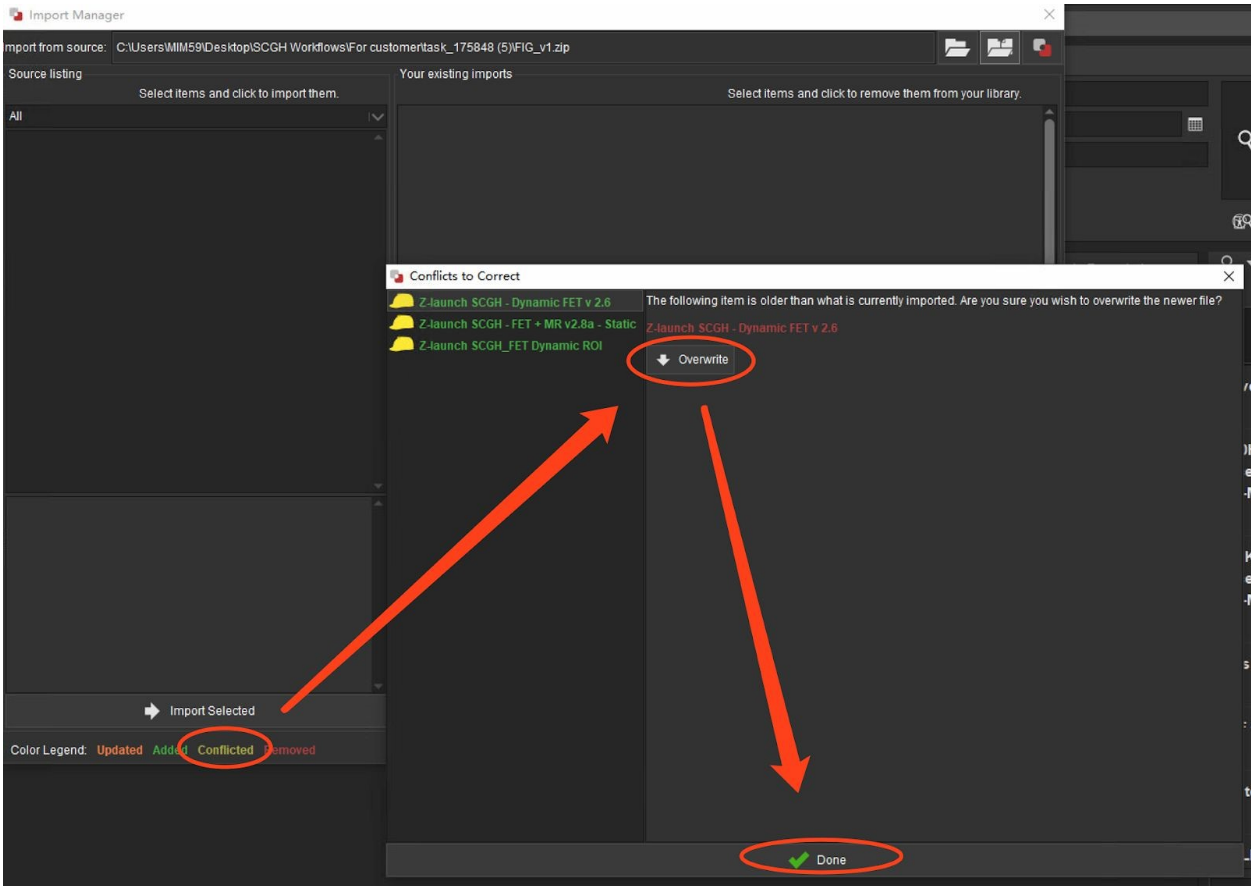

Workflow Running ●

Tech tips:

- While the first time you run the newly updated workflow, you will see a notification like below, just go ahead to Accept Changes to resume the workflow.

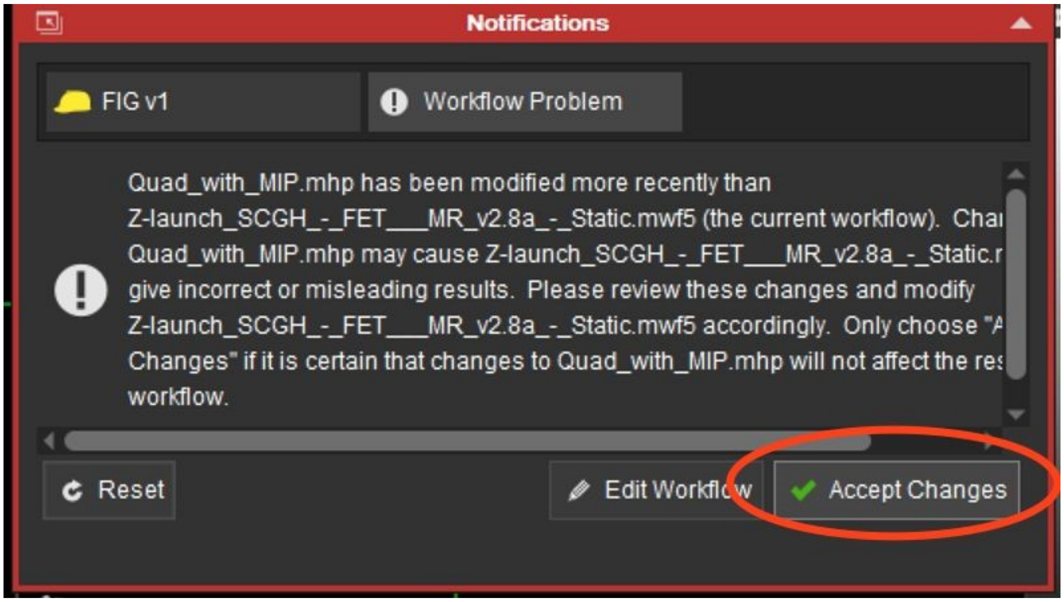

1. Select the patient list in the data source, type the patient name, patient ID or keywords and hit the search button to search the target study. Highlight the dynamic PT, static PT CT, and MR series of the study, toggle on the workflow side panel and search FIG to find the workflow, double click or hit the 'Launch Workflow' button down below to launch the workflow,
    - Tech tips
        - hold the 'Ctrl' button on your keyboard to select multiple series you want.
        - click the little heart icon in the upper right corner of the FIG v1 workflow to
    add it into the Favorites tab for easy access each time. ○ Clinical tips
        - Ideally choose a volumetric MRI series with the highest number of slices (eg: volumetric T1+C).

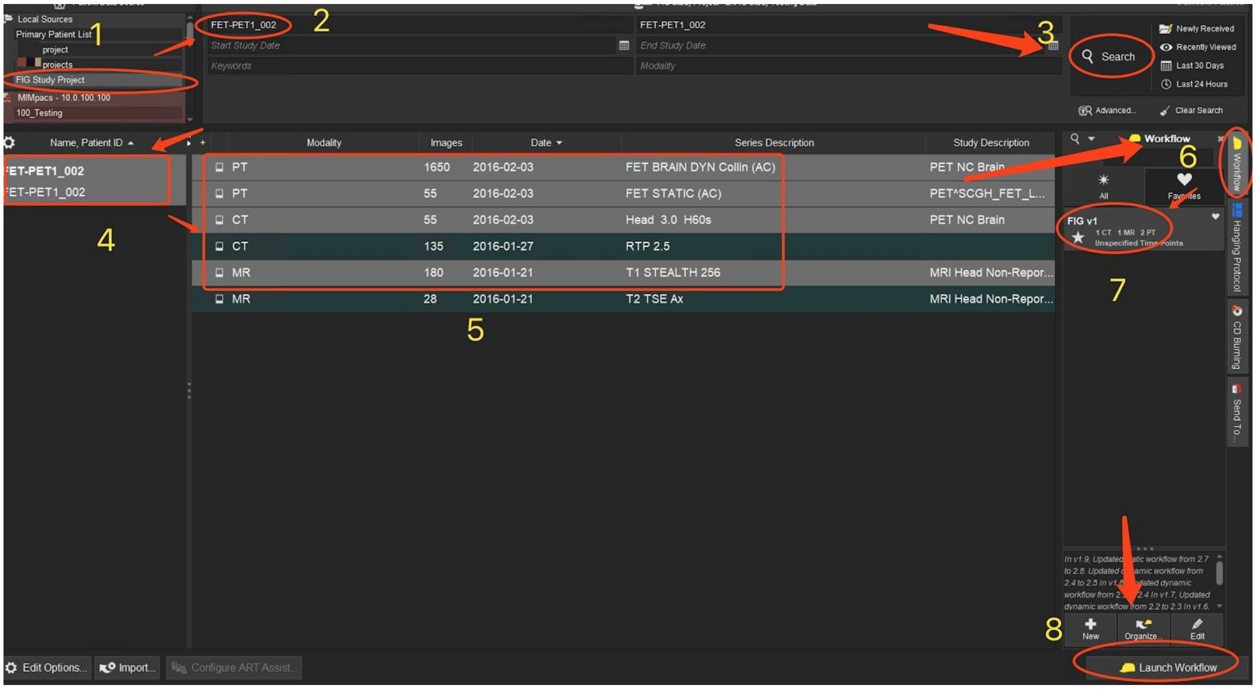

2. Hover your mouse over to the fusion series in the third row of the window, make sure the fusion between the static PT and MR series is ok, then click 'Resume Workflow', ○ Tech tips
        - Use the 'Blend' button to review the fusion. Left click and hold the button up and down to switch between the series.
        - Use the 'Translation/Rotation' buttons to adjust the fusion. Left click and hold the buttons to adjust the static PT series to match the MR series.

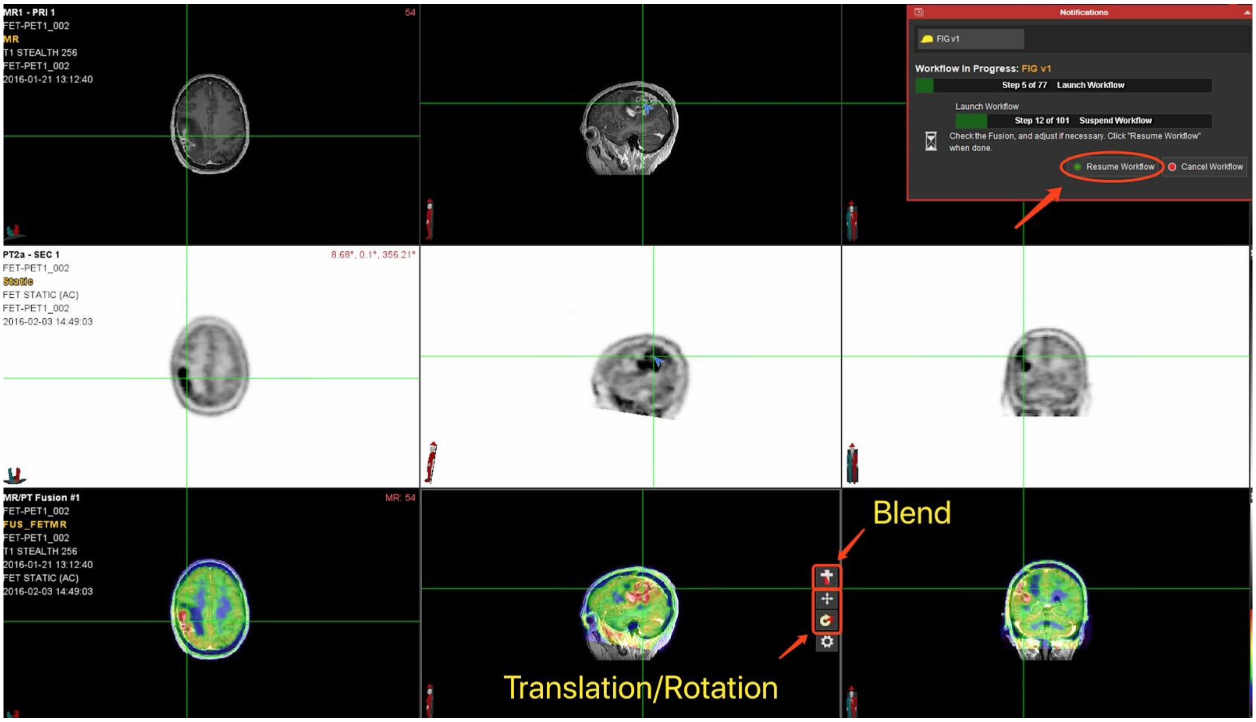

3. Localize to the tumour center, and click 'Resume Workflow', ○ Tech tips
   - ■ Localize to the tumour center in all three planes/viewports,
   - ○ Clinical tips
     - ■ Try to place the localising cross-hairs in the center of the tumour region, even if part of the lesion is necrotic.

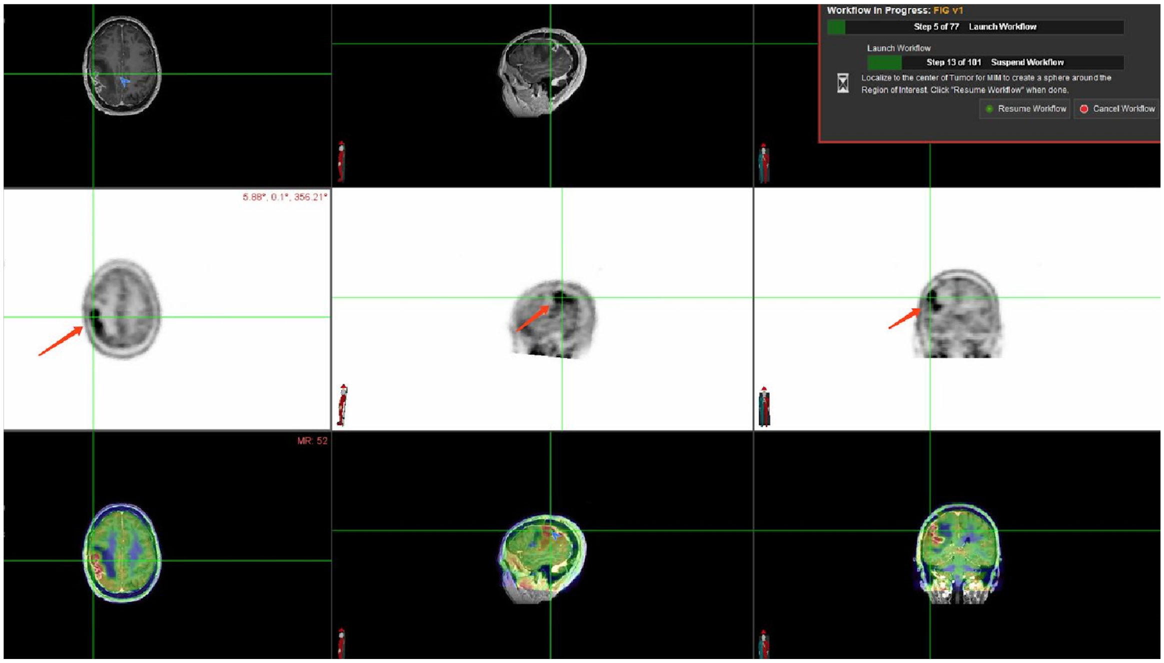

4. Hover your mouse over to the ROI on the Static PT and adjust it if needed to cover the whole tumour, and click ‘Resume Workflow’, ○ Tech tips:
    - Hover your mouse over to the edge of the box surrounding the ROI, when you see the double arrow line, left click and hold to drag to adjust the size of the ROI to cover the tumour completely.
  - Clinical tips:
    - Ensure the entire tumour is encompassed within this large region.

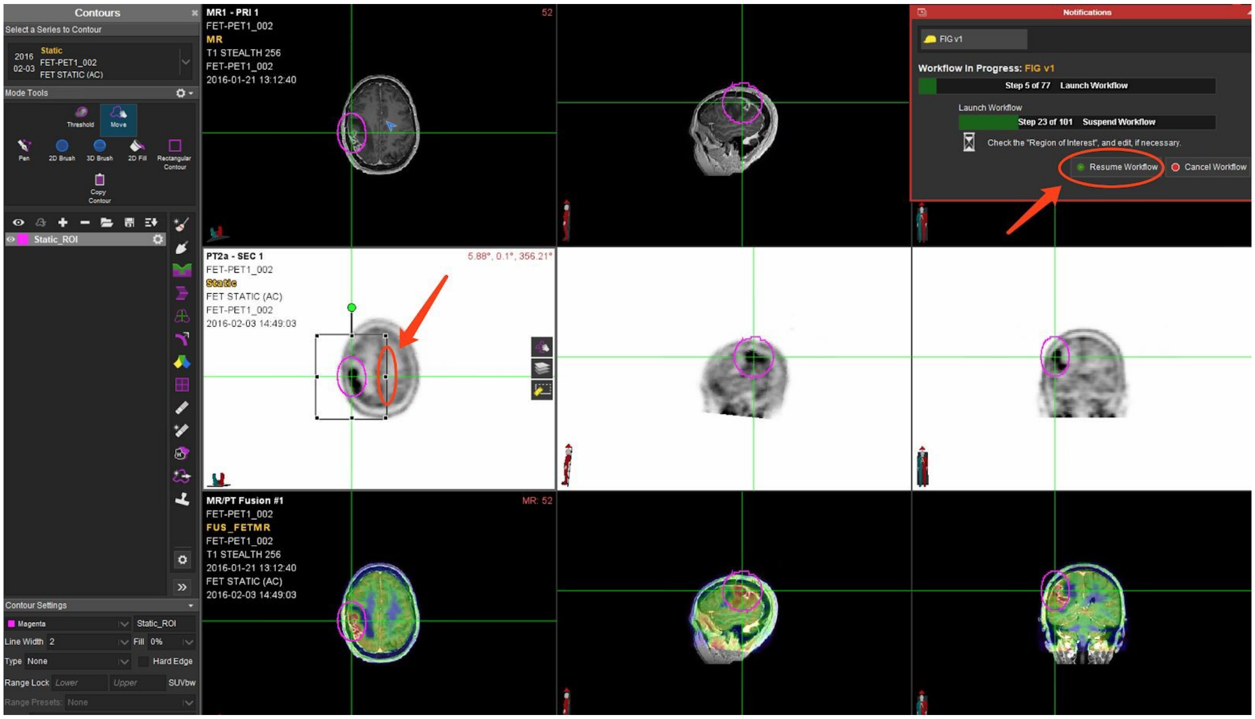

5. Draw the crescent-shaped background, see clinical tips for reference, click ‘Resume Workflow’,
  - Clinical tips:
    - Draw a large crescent shaped background to include the grey/white matter interface extending from anterior to posterior cortex. Place this region in the contralateral cerebral hemisphere in an area with uniform activity, and no oedema/pressure effects or tumour. Ideally place the region at the level of the ventricles.

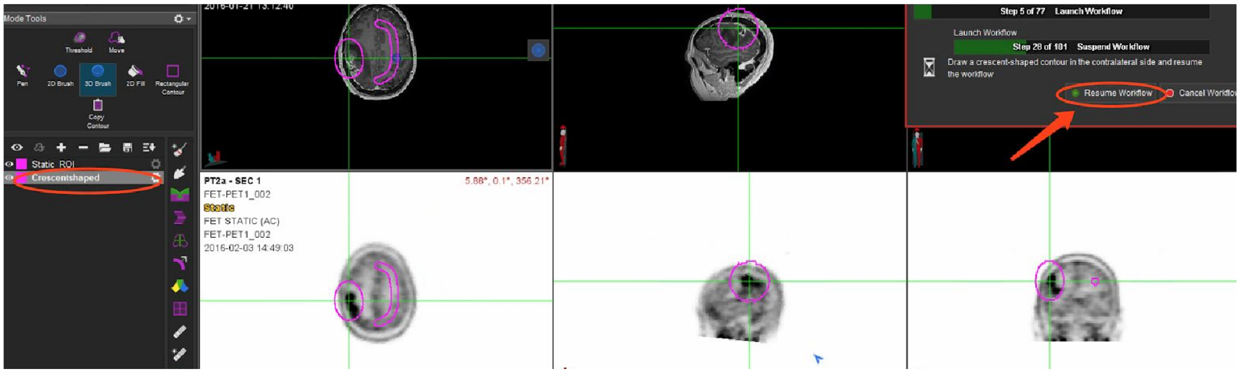

6. Adjust the Static GTV Final contour accordingly, see clinical tips for reference, click 'Resume Workflow', ○ Tech tips:
    - ■ Click the yellow hat in the upper right corner to hide the workflow notification, and click it again to bring it back,
    - ■ Right click and hold up and down to change the size of the 3D brush,
    - ■ Hover your mouse over to any viewport and hit the 'V' button to switch the viewports from Axial to Sagittal to Coronal viewports.
    - ■ Hover your mouse over to any viewport and hit the 'Up' and 'Down'

   buttons to go through slices, ○ Clinical tips:
    - ■ Check the region and edit to ensure all non-tumour areas are deleted (eg: scalp) and any tumour regions which are not part of the automatic region are included. The 3D brush can be used to delete by bringing it towards the region from outside the region, and grow the region by placing the 3D drawing tool inside the region and moving outwards.

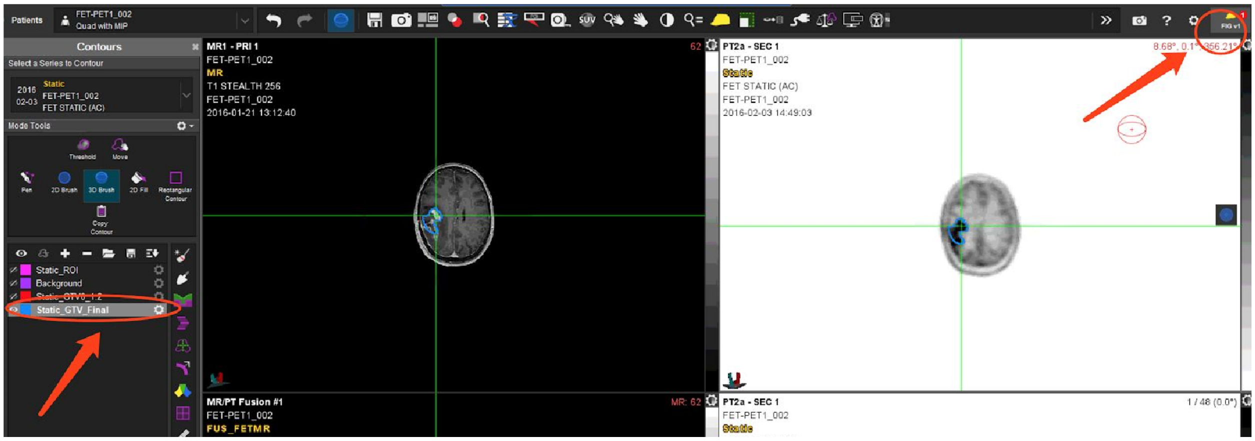

7. Review frames and move the Dynamic Sphere ROI to the desired location on the dynamic image,
    - ○ Tech tips
        - ■ Make sure the Dynamic Sphere is present on the dynamic PT series,
        - ■ You can hover your mouse over to the Dynamic Sphere contour to

   change the location of it if needed, ○ Clinical tips

- Ensure the sphere is located in the tumour. It should be automatically placed in the most intense region of the tumour, but it is important to check the placement.

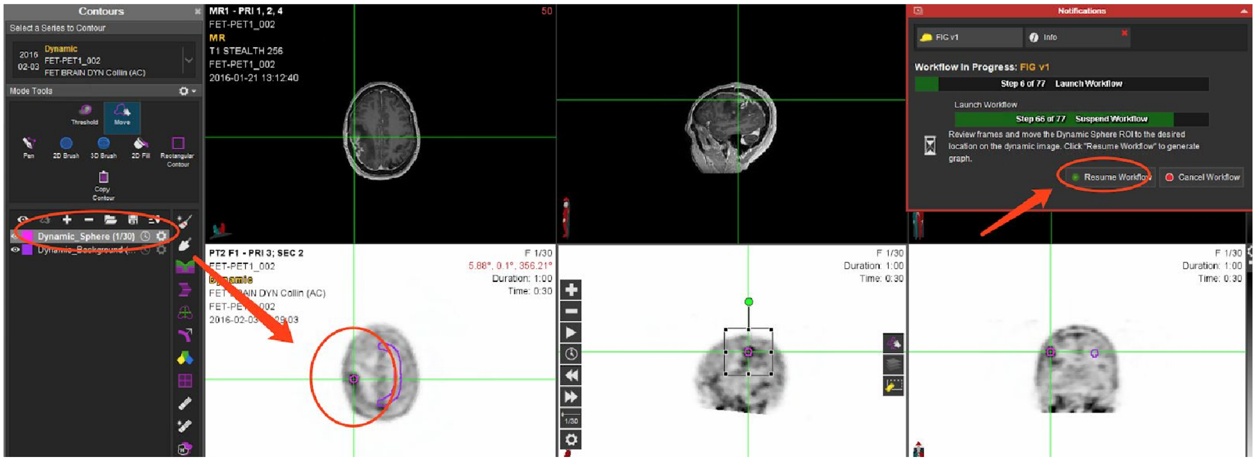

8. Adjust the location of Dynamic Sphere as needed, ○ Tech tips,
   - Hover your mouse over to the viewport and hit the ‘V’ button to switch the viewports in-between axial, sagittal and coronal viewports.
   - Click the left and right double arrow button to go through frames,
   - Hover your mouse over to any viewport and hit the ‘Up’ and ‘Down’ buttons to go through slices, ○ Clinical tips,
   - This step allows you to choose different locations within the area of FET activity to place the dynamic sphere and generate the time activity dynamic curves. This can be useful when trying to ascertain post treatment change from tumour. The final position should ideally be at the site most suggestive of tumour.

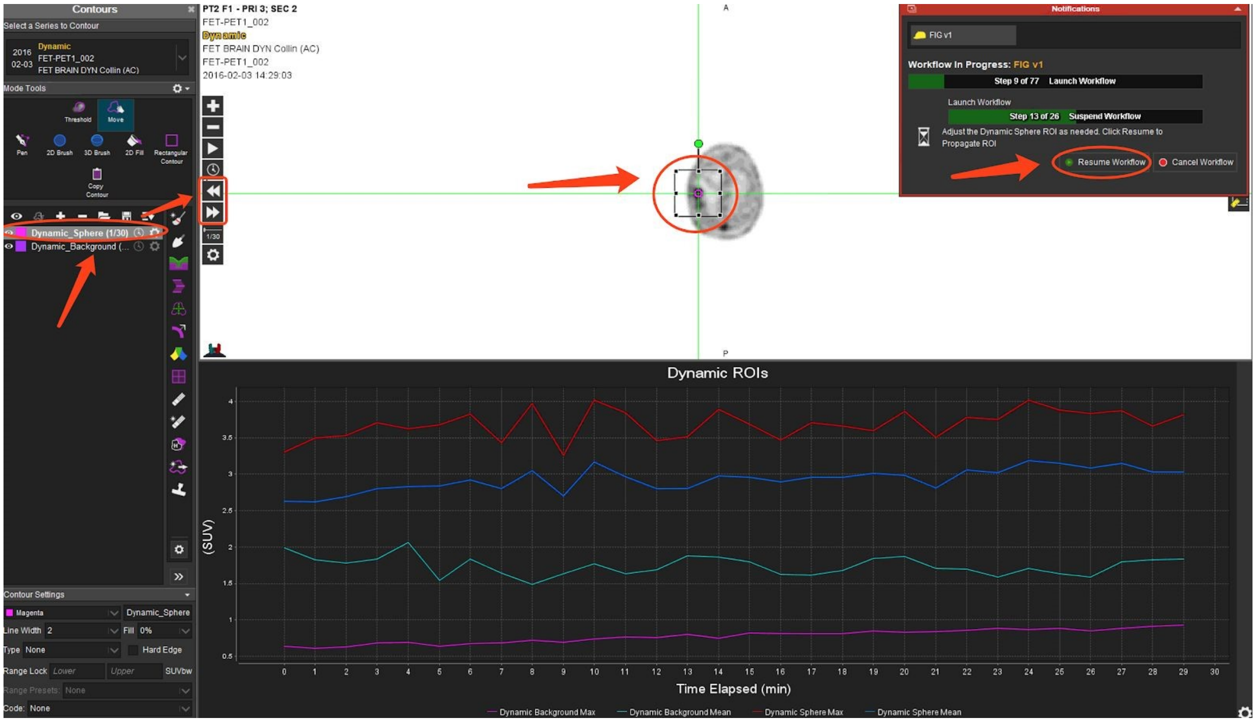

9. Evaluate the propagated contour on all frames and select ‘Yes’ to repeat the last step, or ‘No’ to resume the workflow, ○ Tech tips
   - ■ Click the left and right double arrow button to go through frames,
   - ○ Clinical tips
     - ■ You can review the time activity dynamic curves at this step, and can repeat as often as required to ascertain the dynamic features of different areas of FET activity. This can be useful in determining post treatment change from suspected tumour. The final region chosen will be reflected in the savescreen generated for the workflow as the last step in the analysis.

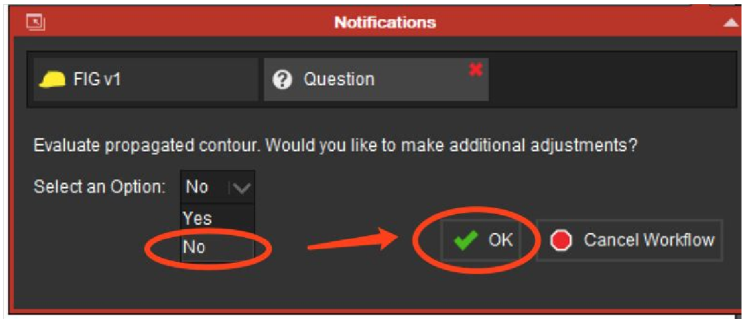

10. Select ‘Yes, save’ to save a session for later review. Review Statistics table and the graph,
    - ○ Tech tips,
      - ■ If this study has already been processed, and one session is saved, you can select ‘No, don’t save’, just for review purposes.

- Hover your mouse over to the viewport and hit the ‘V’ button to switch the viewports in-between axial, sagittal and coronal viewports.
- Click the left and right double arrow button to go through frames,
- Hover your mouse over to any viewport and hit the ‘Up’ and ‘Down’

buttons to go through slices, ○ Clinical tips,

- The statistics listed in the table on this final screen are used to complete the worksheets for the trial.

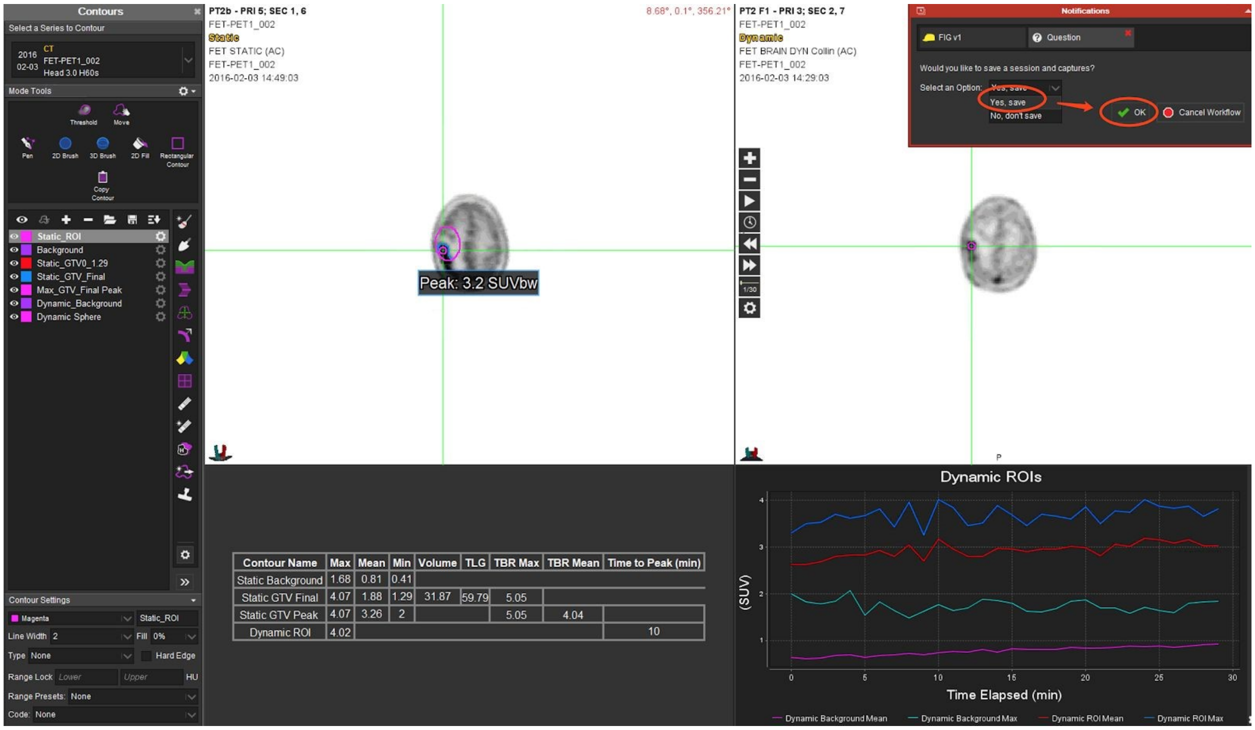

11. Go back to the patient list window, and you’d see the saved RTst, session, and OT series.
    - ○ Tech tips,
        - Session, you can double click it for review purposes.
        - OT, this is the capture of the final page of table and graph. ○

    Clinical tips,

    - The RTst files and OT savescreen need to be sent to TROG (add instructions…. _)

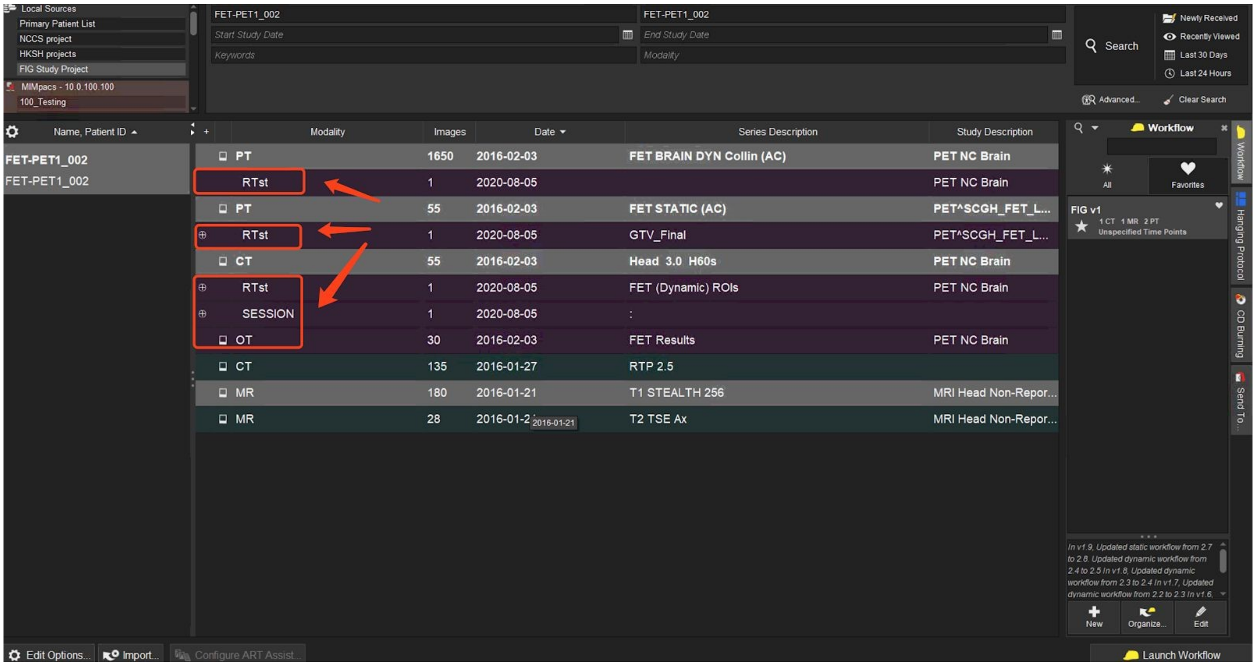

12. Go to the ‘Session’ list in the upper left corner of the screen to close the current session.
    - Tech tips,
        - Select ‘No’ while you see ‘Perform final save of session contours’

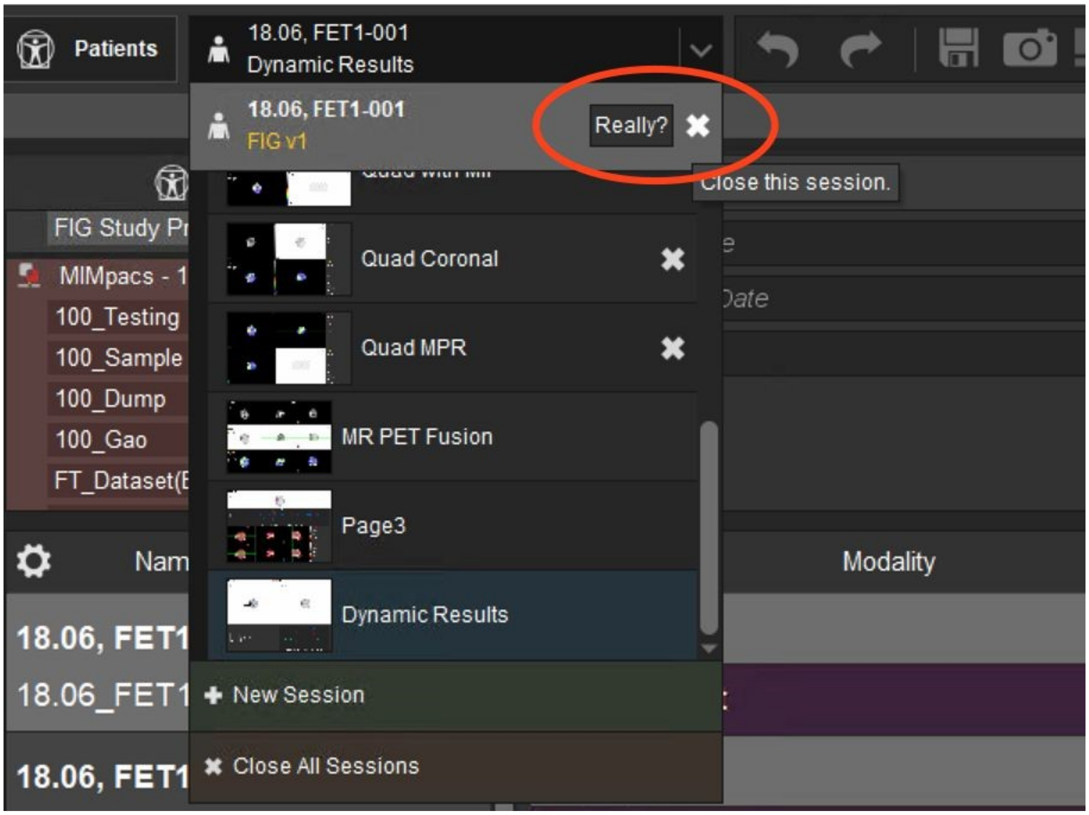

Support

If you have any questions about any step of the workflow, please shoot an email to support@mimsoftware.com, we will provide instant support to help you out.
