# Supplementary material for: Delineation and agreement of FET PET biological volumes in glioblastoma: results of the nuclear medicine credentialing program from the prospective, multi-centre trial evaluating FET PET In Glioblastoma (FIG) study—TROG 18.06
Source: Eur J Nucl Med Mol Imaging. 2023 Aug 11;50(13):3970–81. doi: 10.1007/s00259-023-06371-5 (PMC10611835; doi:10.1007/s00259-023-06371-5)
Supplement: Supplementary file 2 — Supplementary file2 (DOCX 1226 KB) [file 259_2023_6371_MOESM2_ESM.docx]

The European Journal of Nuclear Medicine and Molecular Imaging

**Delineation and agreement of FET PET biological volumes in glioblastoma: results of the Nuclear Medicine credentialing program from the prospective, multi-centre trial evaluating FET PET In Glioblastoma (FIG) Study - TROG 18.06.**

Nathaniel Barry^1,*^, Roslyn J. Francis^2,3^, Martin A. Ebert^1,4^, Eng-Siew Koh^5,6^, Pejman Rowshanfarzad^1^, Ghulam Mubashar Hassan^1^, Jake Kendrick^1^, Hui K. Gan^7,8,9,10^, Sze T. Lee^8,9,10,11^, Eddie Lau^11,12,13^, Bradford A. Moffat^13^, Greg Fitt^12^, Alisha Moore^14^, Paul Thomas^15,16^, David A. Pattison^15,16^, Tim Akhurst^9,17^, Ramin Alipour^9,17^, Elizabeth L. Thomas^2^, Ed Hsiao^18^, Geoffrey P. Schembri^18^, Peter Lin^6,19^, Tam Ly^19^, June Yap^19^, Ian Kirkwood^20,24^, Wilson Vallat^20^, Shahroz Khan^21^, Dayanethee Krishna^21^, Stanley Ngai^22^, Chris Yu^22^, Scott Beuzeville^23^, Tow C. Yeow^23^, Dale Bailey^18,25^, Olivia Cook^14^, Angela Whitehead^14^, Rachael Dykyj^14^, Alana Rossi^14^, Andrew Grose^14^, Andrew M. Scott^8,9,10,11^

*Corresponding Author

1. School of Physics, Mathematics and Computing, University of Western Australia, Crawley, WA, Australia

Email: Nathaniel.barry@research.uwa.edu.au

ORCID ID: 0000-0002-9009-5909

Address: The University of Western Australia, 35 Stirling Highway, Mailbag M013, CRAWLEY, WA 6009

**Credentialling; Classification definitions**

*Acceptable: no variation or variation within tolerance limits.

*Minor/Lesser Deviation: variation that will not have a significant impact on the outcome or interpretation of the study but may require follow-up or education to prevent recurrence in subsequent cases or progression to major deviations.

*Major Deviation: variation from protocol-specified procedures that makes the resulting data questionable and may affect the interpretation of the endpoints.

**Metric definitions:**

Let A and B represent a set of voxels from each respective segmentation:

$$\boldsymbol{DSC}\left( \boldsymbol{A,B} \right)\boldsymbol{=}\frac{\boldsymbol{2(A\cap B)}}{\boldsymbol{A+B}}$$

Let S(Y) denote the set of surface voxels of Y. The shortest distance of a voxel x to S(Y) is defined as:

$$\boldsymbol{d}\left( \boldsymbol{x,S}\left( \boldsymbol{Y} \right) \right)\boldsymbol{=}\min_{\boldsymbol{y}\in S(Y)} \left\| \boldsymbol{x-y} \right\|$$

Where $\left\| . \right\|$ denotes Euclidean distance. Then Hausdorff distance (d_H_) and mean absolute surface distance (MASD) are defined as:

$$\boldsymbol{d}_{\boldsymbol{H}}\left( \boldsymbol{A}\boldsymbol{,}\boldsymbol{B} \right)\boldsymbol{=}\boldsymbol{max}\left\{ \max_{\boldsymbol{a} \in S(A)} \boldsymbol{d}\left( \boldsymbol{a}\boldsymbol{,}\boldsymbol{S}\boldsymbol{(}\boldsymbol{B}\boldsymbol{)} \right)\boldsymbol{,}\max_{\boldsymbol{b} \in S(B)} \boldsymbol{d}\left( \boldsymbol{b}\boldsymbol{,}\boldsymbol{S}\boldsymbol{(}\boldsymbol{A}\boldsymbol{)} \right) \right\}$$

$$\boldsymbol{MASD}\left( \boldsymbol{A,B} \right)\boldsymbol{=}\frac{\boldsymbol{1}}{\boldsymbol{A+B}}\left\{ \sum_{\boldsymbol{a}\in S(A)} \boldsymbol{d(a,S(B))}\boldsymbol{+}\sum_{\boldsymbol{b}\in S(B)} \boldsymbol{d(b,S(A))} \right\}$$

**Table S1** Summary of initial and resubmitted reviews (including retrospective resubmission) of observer contours for all six benchmarking cases. Note that observer 19 was not reviewed. The classifications below relate to BTV delineation for the FET1 cases and response interpretation for the FET3 cases. TAC types were classified for the FET1 cases, but if done incorrectly, did not impact delineation of the final BTV; hence, it was not included in Figure 1 of the manuscript. Furthermore, for FET1, this TAC type error was only made by a single observer who did not classify the TAC type and only provided a numerical value.

| Observer | Case | Acceptable | Minor | Major | Resubmission Required? | Comment (Summary) |
| --- | --- | --- | --- | --- | --- | --- |
| 1 | FET1CASE1 | 1 |  |  |  |  |
| 2 |  |  | 1 |  |  | Background. |
| 3 |  |  |  | 1 | YES | BTV overcontour. |
| 4 |  | 1 |  |  |  |  |
| 5 |  | 1 |  |  |  |  |
| 6 |  |  | 1 |  |  | Background.  BTV undercontour. |
| 7 |  | 1 |  |  |  |  |
| 8 |  |  | 1 |  |  |  |
| 9 |  |  |  | 1 | YES | Background.  TAC pattern. |
| 10 |  | 1 |  |  |  |  |
| 11 |  | 1 |  |  |  |  |
| 12 |  | 1 |  |  |  |  |
| 13 |  | 1 |  |  |  |  |
| 14 |  | 1 |  |  |  |  |
| 15 |  |  | 1 |  | YES | Background. |
| 16 |  | 1 |  |  |  |  |
| 17 |  |  | 1 |  |  | Background.  BTV overcontour. |
| 18 |  | 1 |  |  |  |  |
| 20 |  | 1 |  |  |  |  |
| 21 |  | 1 |  |  |  |  |
| 1 | FET1CASE2 |  | 1 |  |  | BTV undercontour. |
| 2 |  |  |  | 1 | YES | Background. |
| 3 |  |  |  | 1 | YES | BTV overcontour. |
| 4 |  |  |  | 1 | YES | BTV overcontour (scalp). |
| 5 |  |  | 1 |  |  | BTV overcontour (scalp). |
| 6 |  | 1 |  |  |  |  |
| 7 |  | 1 |  |  |  |  |
| 8 |  | 1 |  |  |  |  |
| 9 |  |  |  | 1 | YES | Background.  TAC pattern. |
| 10 |  |  | 1 |  |  |  |
| 11 |  | 1 |  |  |  |  |
| 12 |  |  | 1 |  |  | Background. |
| 13 |  | 1 |  |  |  |  |
| 14 |  |  |  | 1 | YES | BTV overcontour. |
| 15 |  |  |  | 1 | YES | Background.  BTV overcontour (scalp). |
| 16 |  | 1 |  |  |  |  |
| 17 |  |  | 1 |  |  | BTV overcontour. |
| 18 |  | 1 |  |  |  | BTV undercontour. |
| 20 |  |  |  | 1 | YES | Background. |
| 21 |  | 1 |  |  |  |  |
| 1 | FET1CASE3 |  | 1 |  | YES | Background. |
| 2 |  | 1 |  |  |  |  |
| 3 |  |  |  | 1 | YES | BTV overcontour.  Background. |
| 4 |  | 1 |  |  |  |  |
| 5 |  | 1 |  |  |  |  |
| 6 |  | 1 |  |  |  |  |
| 7 |  |  |  |  |  | **NOTE wrong contour. Excluded from analysis.** |
| 8 |  | 1 |  |  |  |  |
| 9 |  |  |  | 1 | YES | TAC pattern. |
| 10 |  | 1 |  |  |  |  |
| 11 |  | 1 |  |  |  |  |
| 12 |  | 1 |  |  |  |  |
| 13 |  | 1 |  |  |  |  |
| 14 |  |  | 1 |  |  |  |
| 15 |  | 1 |  |  |  |  |
| 16 |  | 1 |  |  |  |  |
| 17 |  | 1 |  |  |  |  |
| 18 |  | 1 |  |  |  |  |
| 20 |  | 1 |  |  |  |  |
| 21 |  | 1 |  |  |  |  |
| 1 | FET3CASE1 | 1 |  |  |  | Background.  TAC pattern. |
| 2 |  | 1 |  |  |  |  |
| 3 |  |  |  | 1 | YES | BTV overcontour. |
| 4 |  |  |  | 1 | YES (retro) | Interpretation.  TAC pattern. |
| 5 |  | 1 |  |  |  |  |
| 6 |  | 1 |  |  |  |  |
| 7 |  | 1 |  |  |  |  |
| 8 |  |  | 1 |  |  | Interpretation. |
| 9 |  |  |  | 1 | YES | TAC pattern. |
| 10 |  |  | 1 |  |  | Interpretation. |
| 11 |  | 1 |  |  |  |  |
| 12 |  | 1 |  |  |  | Interpretation. |
| 13 |  | 1 |  |  |  |  |
| 14 |  |  |  | 1 | YES (retro) | Interpretation.  Technical error. |
| 15 |  | 1 |  |  |  |  |
| 16 |  | 1 |  |  |  |  |
| 17 |  |  | 1 |  | YES (retro) | TAC pattern.  Interpretation.  Dynamic sphere volume change. |
| 18 |  | 1 |  |  |  |  |
| 20 |  | 1 |  |  |  | TAC pattern. |
| 21 |  | 1 |  |  |  | Background. |
| 1 | FET3CASE2 | 1 |  |  |  |  |
| 2 |  | 1 |  |  |  |  |
| 3 |  | 1 |  |  | YES | BTV overcontour. |
| 4 |  | 1 |  |  |  |  |
| 5 |  | 1 |  |  |  | TAC pattern. |
| 6 |  | 1 |  |  |  | TAC pattern. |
| 7 |  | 1 |  |  |  |  |
| 8 |  | 1 |  |  |  | Background. |
| 9 |  |  |  | 1 | YES | TAC pattern. |
| 10 |  | 1 |  |  |  |  |
| 11 |  | 1 |  |  |  |  |
| 12 |  | 1 |  |  |  |  |
| 13 |  | 1 |  |  |  |  |
| 14 |  |  | 1 |  | YES (retro) | TAC pattern.  Interpretation. |
| 15 |  | 1 |  |  |  | Background. |
| 16 |  | 1 |  |  |  |  |
| 17 |  | 1 |  |  | YES | TAC pattern.  BTV overcontour.  Dynamic sphere volume. |
| 18 |  | 1 |  |  |  |  |
| 20 |  | 1 |  |  |  |  |
| 21 |  | 1 |  |  |  | Background. |
| 1 | FET3CASE3 | 1 |  |  |  | BTV undercontour. |
| 2 |  | 1 |  |  |  | Background. |
| 3 |  | 1 |  |  | YES | BTV overcontour. |
| 4 |  | 1 |  |  | YES (retro) | BTV overcontour (scalp). |
| 5 |  | 1 |  |  |  |  |
| 6 |  | 1 |  |  |  |  |
| 7 |  | 1 |  |  |  | BTV overcontour (scalp). |
| 8 |  |  | 1 |  |  | TAC pattern. |
| 9 |  |  |  | 1 | YES | TAC pattern. |
| 10 |  |  | 1 |  |  | Interpretation. |
| 11 |  | 1 |  |  |  |  |
| 12 |  | 1 |  |  |  |  |
| 13 |  | 1 |  |  |  |  |
| 14 |  |  |  | 1 | YES (retro) | TAC pattern.  Interpretation. |
| 15 |  |  |  | 1 | YES | BTV overcontour (scalp).  TAC pattern.  Interpretation. |
| 16 |  | 1 |  |  |  | BTV undercontour. |
| 17 |  | 1 |  |  |  |  |
| 18 |  | 1 |  |  |  |  |
| 20 |  | 1 |  |  |  |  |
| 21 |  |  | 1 |  |  | Interpretation. |

RESUB1

| Observer | Case | Acceptable | Minor | Major | Resub | Comment |
| --- | --- | --- | --- | --- | --- | --- |
| 3 | FET1CASE1 | 1 |  |  |  |  |
| 9 |  | 1 |  |  |  |  |
| 15 |  | 1 |  |  |  |  |
| 2 | FET1CASE2 | 1 |  |  |  |  |
| 3 |  |  | 1 |  |  |  |
| 4 |  |  |  | 1 | YES | BTV overcontour (scalp). |
| 9 |  | 1 |  |  |  |  |
| 14 |  | 1 |  |  |  |  |
| 15 |  | 1 |  |  |  |  |
| 20 |  | 1 |  |  |  |  |
| 3 | FET1CASE3 | 1 |  |  |  |  |
| 9 |  | 1 |  |  |  |  |
| 3 | FET3CASE1 |  | 1 |  | YES | BTV overcontour (scalp+sagittal sinus). |
| 9 |  |  | 1 |  |  | Interpretation. |
| 14 |  | 1 |  |  |  |  |
| 17 |  |  | 1 |  |  |  |
| 3 | FET3CASE2 |  | 1 |  | YES | BTV overcontour.  Interpretation. |
| 9 |  |  | 1 |  |  |  |
| 14 |  | 1 |  |  |  |  |
| 17 |  | 1 |  |  |  |  |
| 3 | FET3CASE3 | 1 |  |  |  |  |
| 9 |  |  | 1 |  |  | Interpretation. |
| 14 |  | 1 |  |  |  |  |
| 15 |  | 1 |  |  |  |  |

RESUB2

| Observer | Case | Acceptable | Minor | Major | Resub | Comment |
| --- | --- | --- | --- | --- | --- | --- |
| 4 | FET1CASE2 | 1 |  |  |  |  |
| 3 | FET3CASE1 |  | 1 |  |  | BTV overcontour (scalp).  TAC pattern.  Interpretation. |
| 3 | FET3CASE2 | 1 |  |  |  |  |
| FET1 TOTAL |  | 47 | 13 | 12 | 14 |  |
| FET3 TOTAL |  | 52 | 14 | 8 | 16 |  |

**Table S2** Mean, standard deviation (SD), and coefficient of variation (COV) of observer contour volumes.

| Structure | Case | FET1CASE1 | FET1CASE2 | FET1CASE3 | FET3CASE1 | FET3CASE2 | FET3CASE3 | ALL |
| --- | --- | --- | --- | --- | --- | --- | --- | --- |
| BTV | mean | 34.13 | 64.69 | 17.99 | 44.17 | 68.32 | 14.78 | 40.86 |
|  | SD | 8.13 | 7.76 | 4.29 | 8.4 | 20.56 | 2.84 | 23.19 |
|  | COV | 23.83 | 12.0 | 23.87 | 19.01 | 30.1 | 19.23 | 56.76 |
| GTV0 | mean | 32.35 | 65.74 | 17.86 | 42.59 | 69.0 | 17.58 | 41.04 |
|  | SD | 6.64 | 8.93 | 5.07 | 7.34 | 19.94 | 5.93 | 23.07 |
|  | COV | 20.53 | 13.58 | 28.39 | 17.23 | 28.89 | 33.74 | 56.21 |
| Static VOI | mean | 152.58 | 181.97 | 68.37 | 146.79 | 158.08 | 198.89 | 151.78 |
|  | SD | 51.02 | 44.15 | 32.59 | 43.74 | 98.5 | 69.0 | 73.05 |
|  | COV | 33.44 | 24.26 | 47.66 | 29.8 | 62.31 | 34.69 | 48.13 |

**Table S3** Distribution of pairwise Dice Similarity Coefficients.

| Structure | Case | FET1CASE1 | FET1CASE2 | FET1CASE3 | FET3CASE1 | FET3CASE2 | FET3CASE3 | ALL |
| --- | --- | --- | --- | --- | --- | --- | --- | --- |
| BTV | mean | 0.85 | 0.88 | 0.85 | 0.85 | 0.77 | 0.84 | 0.84 |
|  | SD | 0.08 | 0.05 | 0.09 | 0.08 | 0.09 | 0.08 | 0.09 |
|  | COV | 9.72 | 5.36 | 10.29 | 9.03 | 11.64 | 9.7 | 10.29 |
| GTV0 | mean | 0.87 | 0.88 | 0.85 | 0.88 | 0.79 | 0.76 | 0.84 |
|  | SD | 0.07 | 0.05 | 0.1 | 0.06 | 0.09 | 0.11 | 0.1 |
|  | COV | 8.28 | 5.98 | 12.25 | 7.35 | 11.61 | 14.09 | 11.59 |
| Static VOI | mean | 0.71 | 0.81 | 0.7 | 0.76 | 0.66 | 0.69 | 0.72 |
|  | SD | 0.1 | 0.08 | 0.13 | 0.09 | 0.16 | 0.17 | 0.13 |
|  | COV | 13.79 | 9.69 | 18.73 | 11.87 | 23.9 | 23.87 | 18.57 |

**Table S4** Distribution of pairwise Hausdorff distances.

| Structure | Case | FET1CASE1 | FET1CASE2 | FET1CASE3 | FET3CASE1 | FET3CASE2 | FET3CASE3 | ALL |
| --- | --- | --- | --- | --- | --- | --- | --- | --- |
| BTV | mean | 15.14 | 12.78 | 10.15 | 14.59 | 23.37 | 18.09 | 15.78 |
|  | SD | 7.84 | 2.74 | 5.65 | 6.25 | 9.4 | 8.89 | 8.3 |
|  | COV | 51.76 | 21.45 | 55.69 | 42.81 | 40.22 | 49.14 | 52.6 |
| GTV0 | mean | 16.42 | 15.1 | 11.22 | 19.22 | 20.04 | 27.78 | 18.41 |
|  | SD | 8.46 | 4.44 | 6.94 | 5.03 | 8.91 | 7.32 | 8.69 |
|  | COV | 51.49 | 29.43 | 61.84 | 26.15 | 44.47 | 26.36 | 47.2 |
| Static VOI | mean | 17.35 | 11.82 | 12.43 | 13.88 | 19.45 | 18.25 | 15.58 |
|  | SD | 7.05 | 4.21 | 4.05 | 4.79 | 10.27 | 6.87 | 7.24 |
|  | COV | 40.65 | 35.58 | 32.58 | 34.48 | 52.8 | 37.67 | 46.45 |


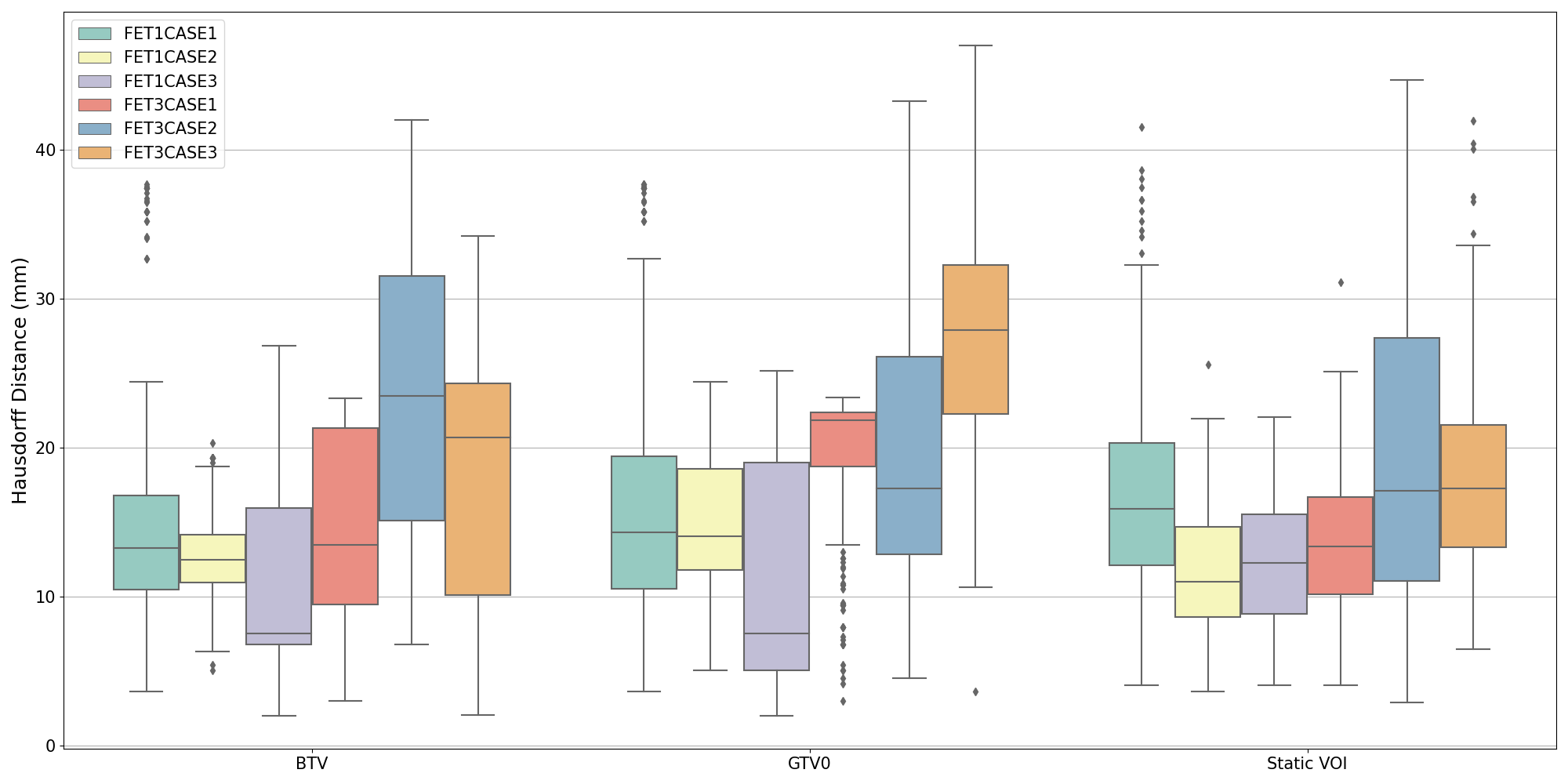


**Fig. S1** Pairwise Hausdorff Distance scores grouped by case.

**Table S5** Distribution of pairwise Mean Absolute Surface Distances.

| Structure | Case | FET1CASE1 | FET1CASE2 | FET1CASE3 | FET3CASE1 | FET3CASE2 | FET3CASE3 | ALL |
| --- | --- | --- | --- | --- | --- | --- | --- | --- |
| BTV | mean | 1.24 | 0.77 | 1.09 | 1.1 | 3.41 | 1.2 | 1.47 |
|  | SD | 0.82 | 0.34 | 0.78 | 0.68 | 1.95 | 0.77 | 1.36 |
|  | COV | 65.62 | 44.55 | 71.21 | 62.01 | 57.32 | 63.89 | 91.93 |
| GTV0 | mean | 1.09 | 0.81 | 1.54 | 1.18 | 3.12 | 3.22 | 1.83 |
|  | SD | 0.84 | 0.43 | 1.54 | 0.7 | 2.06 | 2.19 | 1.76 |
|  | COV | 77.4 | 52.62 | 100.13 | 59.44 | 66.13 | 67.83 | 95.96 |
| Static VOI | mean | 5.68 | 3.76 | 4.61 | 4.69 | 7.41 | 6.04 | 5.38 |
|  | SD | 2.11 | 1.71 | 2.58 | 1.99 | 4.79 | 2.78 | 3.09 |
|  | COV | 37.26 | 45.38 | 55.95 | 42.46 | 64.63 | 45.98 | 57.45 |


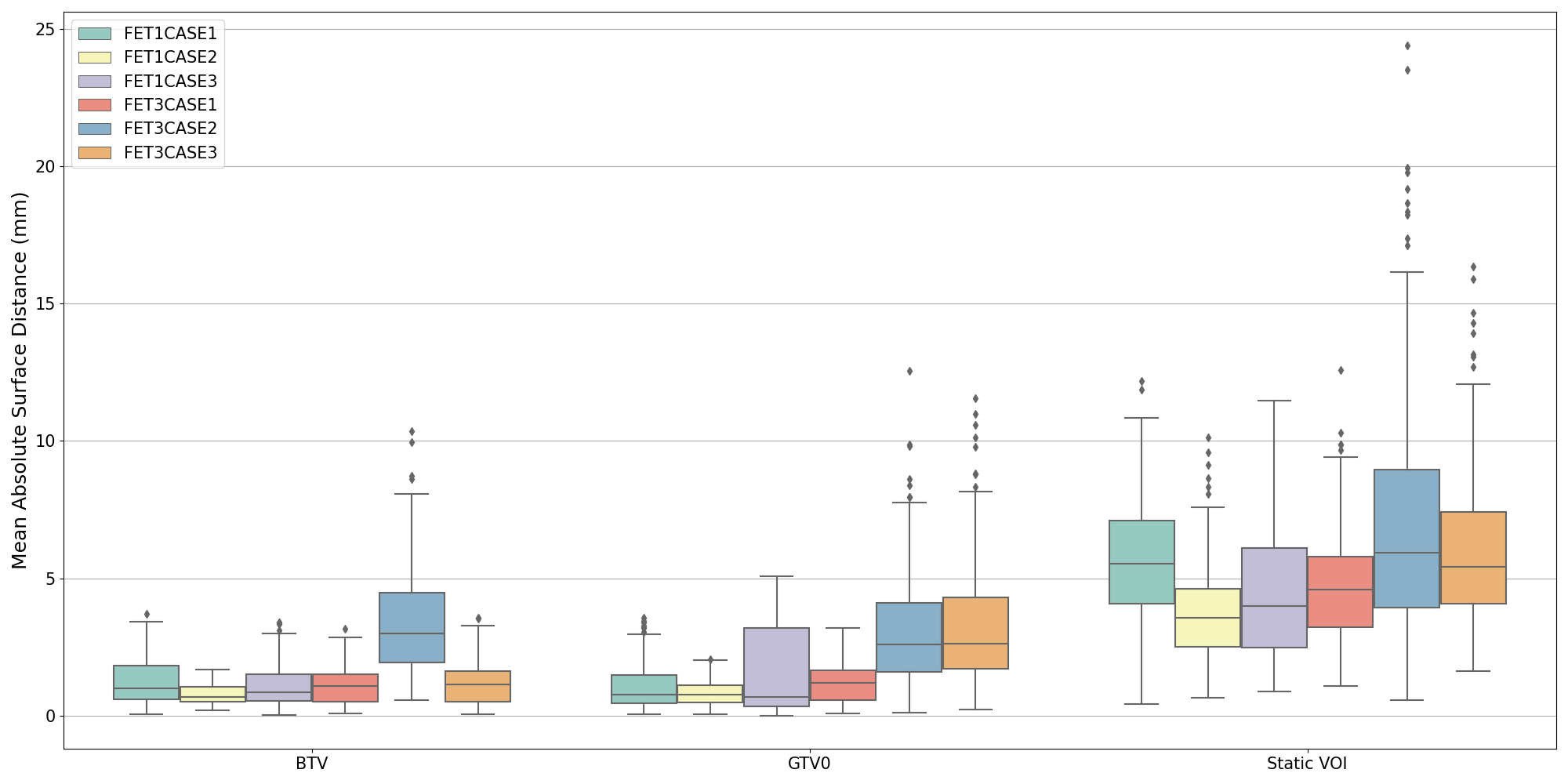


**Fig. S2** Pairwise Mean Absolute Surface Distance scores grouped by case.

**Table S6** Mean, standard deviation (SD), and coefficient of variation (COV) of SUVmean.

| Structure | Case | FET1CASE1 | FET1CASE2 | FET1CASE3 | FET3CASE1 | FET3CASE2 | FET3CASE3 | ALL |
| --- | --- | --- | --- | --- | --- | --- | --- | --- |
| Background | mean | 0.9 | 1.08 | 1.07 | 1.18 | 1.27 | 0.96 | 1.08 |
| Background | SD | 0.06 | 0.05 | 0.07 | 0.06 | 0.07 | 0.06 | 0.14 |
| Background | COV | 6.14 | 4.78 | 6.32 | 5.22 | 5.49 | 6.12 | 12.87 |


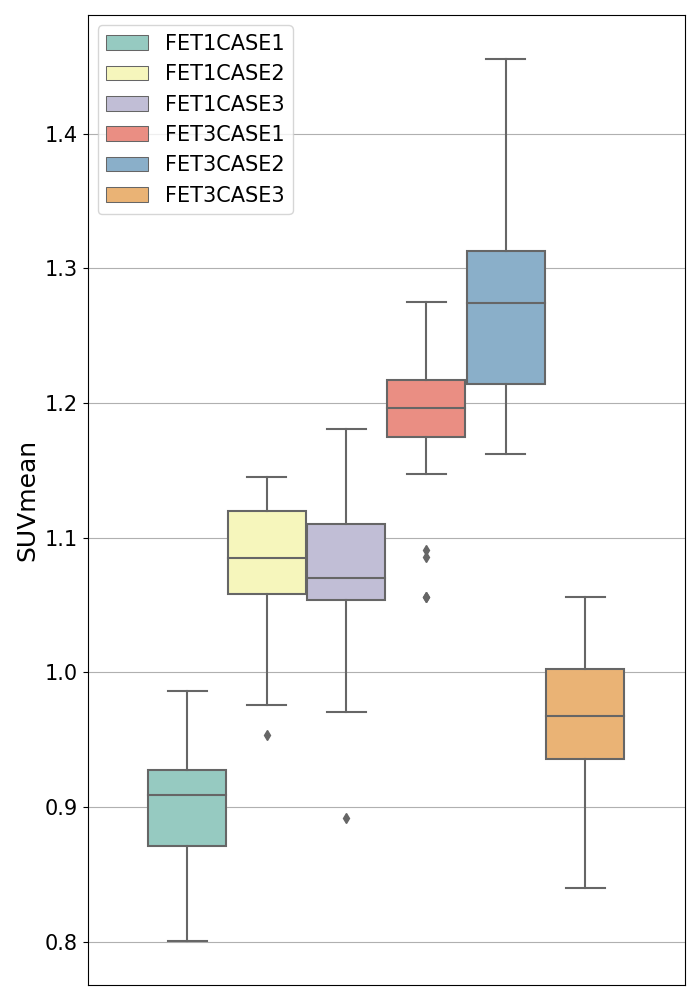


**Fig. S3** Distribution of SUVmean grouped by case.


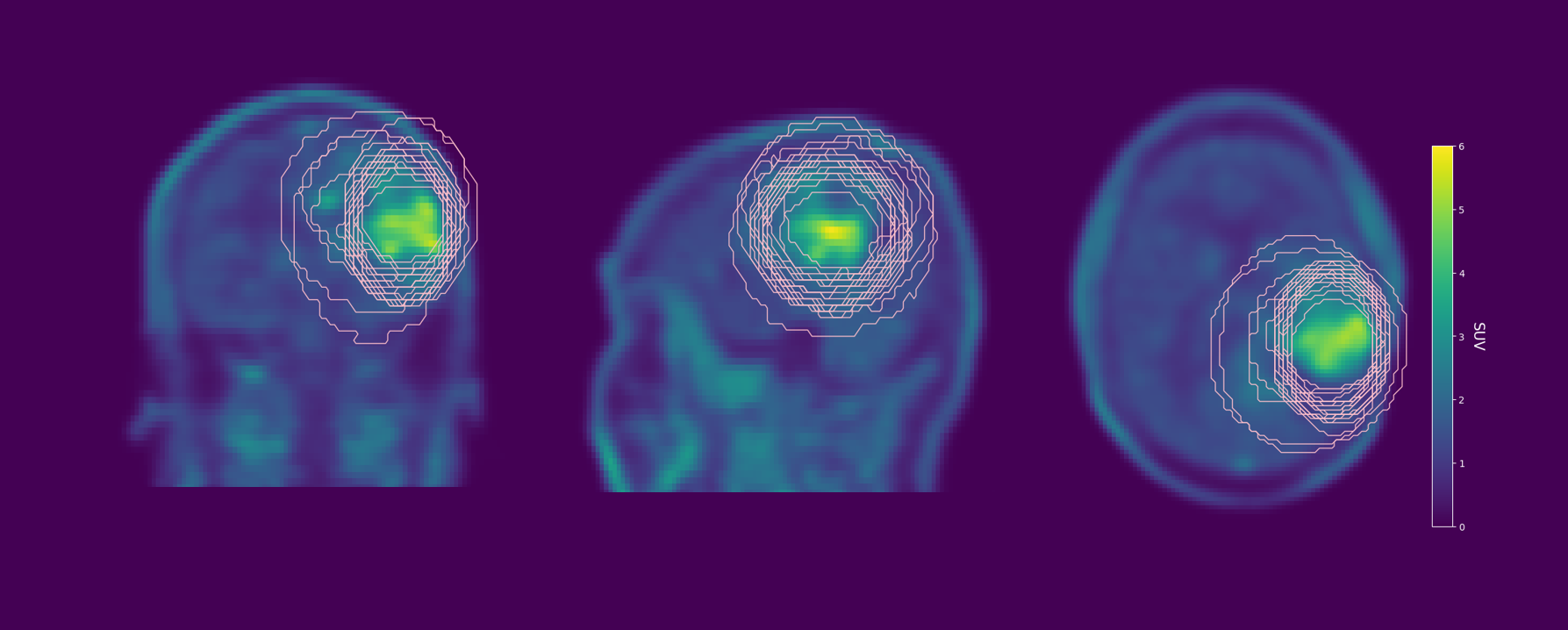


**Fig. S4** All Static VOI contours superimposed (pink) over FET3CASE2. Notice how the larger initial spheres overlap with the additional area of uptake nearer to the midline which would then be incorporated into the final BTV.

**
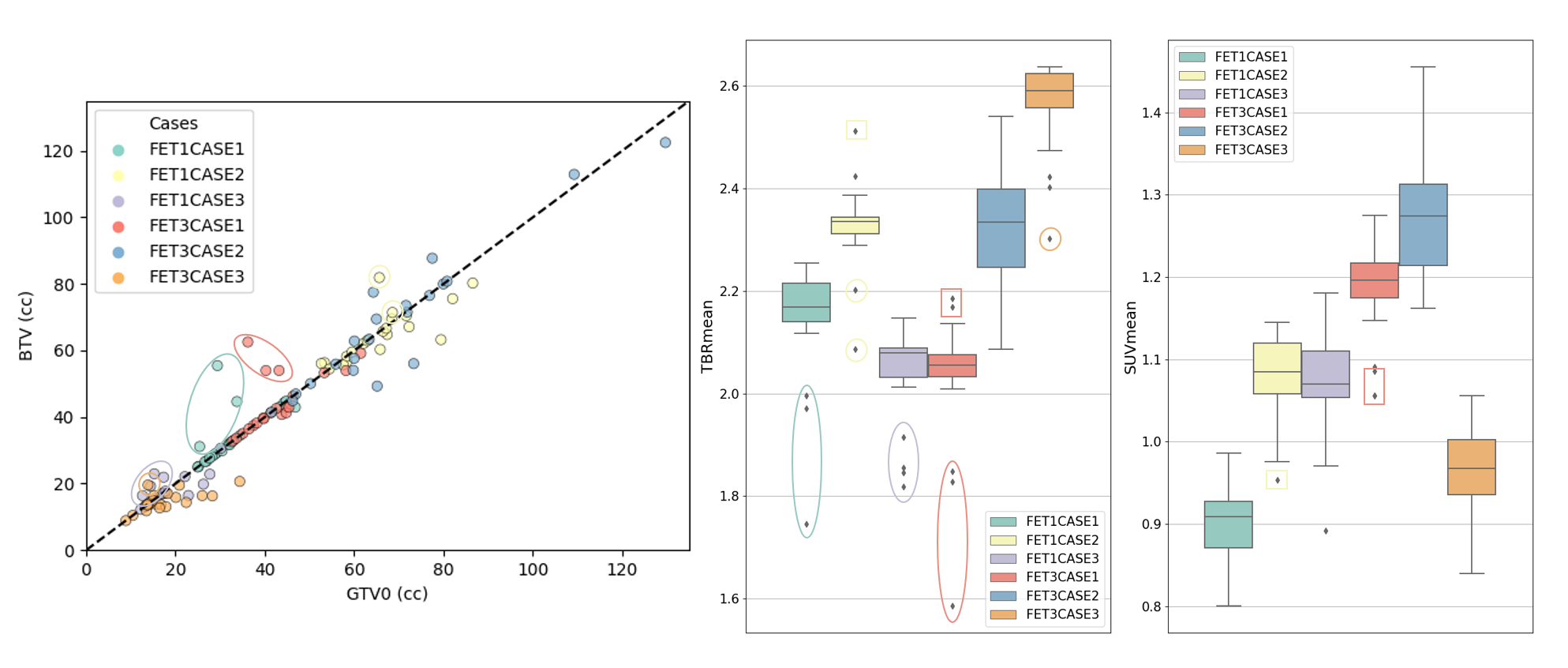
**

**Fig. S5** Investigating the cause of TBRmean outliers. A scatter plot (left) shows any change in volume after thresholding (GTV0) when manual adjustment is done to give the final BTV. Dots above the line of equivalence (dashed line) indicate an addition of volume and those below indicated a reduction. The matching circles show that adding volume often caused an outlier TBRmean below the median (middle boxplot). The volume of the remaining two outliers in FET3CASE3 below the median did not change significantly. Further investigation found that the addition of volume was compensated by the removal of volume elsewhere, but observers still manually included areas of low uptake, leading to the observed drop in TBRmean. TBRmean outliers above the median are illustrated by the matching squares with SUVmean outliers (right boxplot). The lower SUVmean causes the TBR of every voxel within the BTV to increase; hence, an increase in TBRmean. Lastly, the outlier SUVmean in FET1CASE3 did not cause an outlier TBRmean as areas of low uptake were manually included. Although, it was the highest TBRmean.


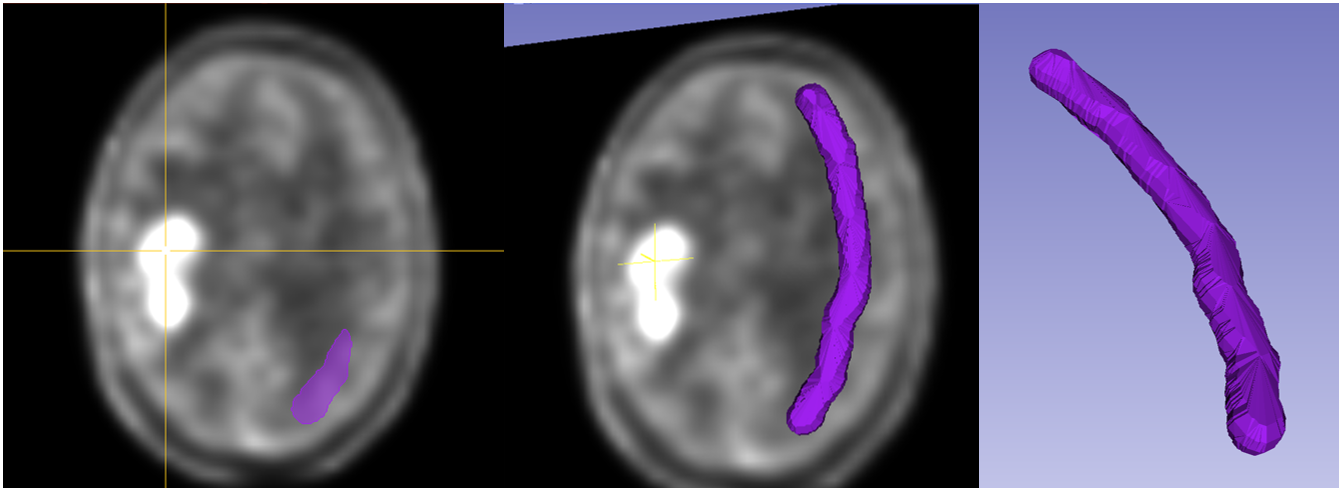


**Fig. S6** Example to illustrate the shape of the background contour. Note that when registered to PET space, the background contour passes through multiple axial slices; hence, the small amount of contour shown on the left.
